# Anti-Neurodegenerating Activity: Structure–Activity Relationship Analysis of Flavonoids

**DOI:** 10.3390/molecules28207188

**Published:** 2023-10-20

**Authors:** Gagan Preet, Ahlam Haj Hasan, Piteesha Ramlagan, Shameem Fawdar, Fabien Boulle, Marcel Jaspars

**Affiliations:** 1 Marine Biodiscovery Centre, Department of Chemistry, University of Aberdeen, Aberdeen AB24 3UE, UK; gagan.preet1@abdn.ac.uk (G.P.); a.hajhasan.20@abdn.ac.uk (A.H.H.); 2 Department of Medicinal Chemistry and Pharmacognosy, Faculty of Pharmacy, Jordan University of Science and Technology, Irbid 22110, Jordan; 3 Axonova Ltd., Grand Port 51405, Mauritius; p.ramlagan@axonova-pharma.com (P.R.); s.fawdar@axonova-pharma.com (S.F.); f.boulle@axonova-pharma.com (F.B.)

**Keywords:** flavones, flavonoids, SAR, neurodegeneration, computational, in silico

## Abstract

An anti-neurodegeneration activity study was carried out for 80 flavonoid compounds. The structure–activity analysis of the structures was carried out by performing three different anti-neurodegeneration screening tests, showing that in these structures, the presence of a hydroxy substituent group at position C3′ as well as C5′ of ring B and a methoxy substituent group at the C7 position of ring A play a vital role in neuroprotective and antioxidant as well as anti-inflammatory activity. Further, we found structure (**5**) was the top-performing active structure out of 80 structures. Subsequently, a molecular docking study was carried out for the 3 lead flavonoid compounds (**4**), (**5**), and (**23**) and 21 similar hypothetical proposed structures to estimate the binding strength between the tested compounds and proteins potentially involved in disease causation. Ligand-based pharmacophores were generated to guide future drug design studies.

## 1. Introduction

Almost two decades ago, Przedborski et al. [1] reported an absolute analysis of anti-neurodegenerative activity that is still highly relevant today. They described neurodegeneration principally as “any pathological condition predominantly affecting neurons”. More precisely, they called neurodegenerative diseases a massive, heterogeneous combination of neurological ataxia (disorders that affect coordination, balance, and speech) that influence specific subsets of neurons in distinct anatomical regions. Among the neurodegenerative disorders, Alzheimer’s disease (AD), Parkinson’s disease (PD), Huntington’s disease (HD), and amyotrophic lateral sclerosis (ALS) are the most researched [2]. According to a report in the United States, 5.8 million people suffer from AD at present [3], over 700,000 with PD [4], ~30,000 with HD [5], ~16,000 with ALS [6], and 50,000–60,000 with frontotemporal disorders (FTD) [2]. 

Increasing age is known to be the biggest risk factor for developing neurodegenerative diseases. This indicates that modifications that arise during ageing increase the risk of neurodegenerative diseases. Identifying those modifications could provide a route to establish therapeutics that can at least reduce, if not prevent, disease development or advancement.

Neurodegenerative disorders are also manifested by diverse clinical symptoms, including memory and cognitive impairment, breathing problems, speaking disability, and motor dysfunction [7,8,9,10,11]. These symptoms are outcomes of stress conditions that affect normal cell function, such as oxidative stress, environmental stress (e.g., metals and pesticides), and pharmaceuticals [12,13]. Oxidative stress in the brain increases with aging, and it is considered a risk factor for developing Alzheimer’s disease [14,15,16,17]. Oxidative stress is caused by a redox state imbalance, which results from the excess formation of reactive oxygen species (ROS) and/or the dysfunction of the antioxidant system. ROS significantly deteriorate neuronal cells via targeting different biomolecules in the cells, such as DNA, lipids, and proteins, and affecting their functions [14,15,16,17]. Also, the role of tumour necrosis factor α (TNF-α) as a neurotoxic agent in the pathogenesis of AD has been confirmed in clinical and animal studies, and researchers are continuing to seek TNF inhibitors that can cross the BBB, as the available TNF inhibitors on the market have no BBB permeability [18,19]. 

AD is complex and multifactorial by nature, and the exact root cause of this disease is not understood [20]. According to the World Health Organization, AD is one of the most common universal neurodegenerative diseases. The World Alzheimer Report 2019 predicted that over 50 million people have dementia globally, which is set to rise to 152 million by 2050 [21] due to a lack of adequate treatment options. The clinical indications of AD are non-functioning neurons and progressive neuronal death, followed by progressive memory loss and cognitive failure related to the loss of cholinergic and glutamatergic processes [22]. Discrete enzyme targets have been identified, comprising cholinesterases such as acetylcholinesterase (AChE), which hydrolyses acetylcholine, and butyrylcholinesterase (BuChE), which acts as a co-regulator of the acetylcholine level and affects beta-secretase, glycogen synthase kinase-3b, and NADPH, which play vital roles in the pathogenesis of AD [23]. 

AD is associated with a deficit of the cholinergic system, with reduced levels of acetylcholine (ACh) in the region of the brain responsible for emotion, memory, behaviour, and learning [20,21]. One way to treat AD symptoms is to increase the synaptic levels of ACh in the brain by blocking the acetylcholinesterase (AChE) enzyme, which binds and hydrolyses ACh. Galanthamine, donepezil, rivastigmine, and tacrine are the main AChE inhibitors used clinically to control AD [24,25,26,27,28,29,30]. 

Neuropathologically, AD is indicated by the presence of β-amyloid (Aβ) plaques in distinct brain regions, as well as intraneuronal insoluble neurofibrillary tangles (NFTs) composed of Tau protein [26,27]. Aβ and NFTs are the major factors in AD progression [27]. Numerous therapeutic approaches towards AD control have focused on reducing Aβ aggregation by inhibiting BACE-1, which is involved in Aβ generation, but this approach has been shown not to work. 

Glycogen synthase kinase-3b (GSK-3b) is a significant kinase (tau kinase) involved in tau phosphorylation, and its function is involved in countless illnesses [31]. Cyclic adenosine monophosphate (cAMP) is known to decrease neuroinflammation and is responsible for memory formation and cognition. The phosphodiesterase 4B enzyme (PDE4B) is responsible for the hydrolysis of cyclic (AMP). Thus it is considered an excellent therapeutic target that may decrease the neuroinflammation in AD [32,33,34]. An in vitro study showed that increasing the cAMP level enhances neuron growth [35].

As mentioned above, oxidative stress is one of the primary factors in the pathological area, embroiled in the pathogenesis of numerous diseases, including AD [36]. In AD, Aβ upregulates NADPH oxidase activity and escalates the oxidative stress. Consequently, the suppression of NADPH oxidase or NADPH oxidase-mediated inflammatory and oxidative stress factor has been attempted to regulate AD [37,38]. 

**Figure 1:**
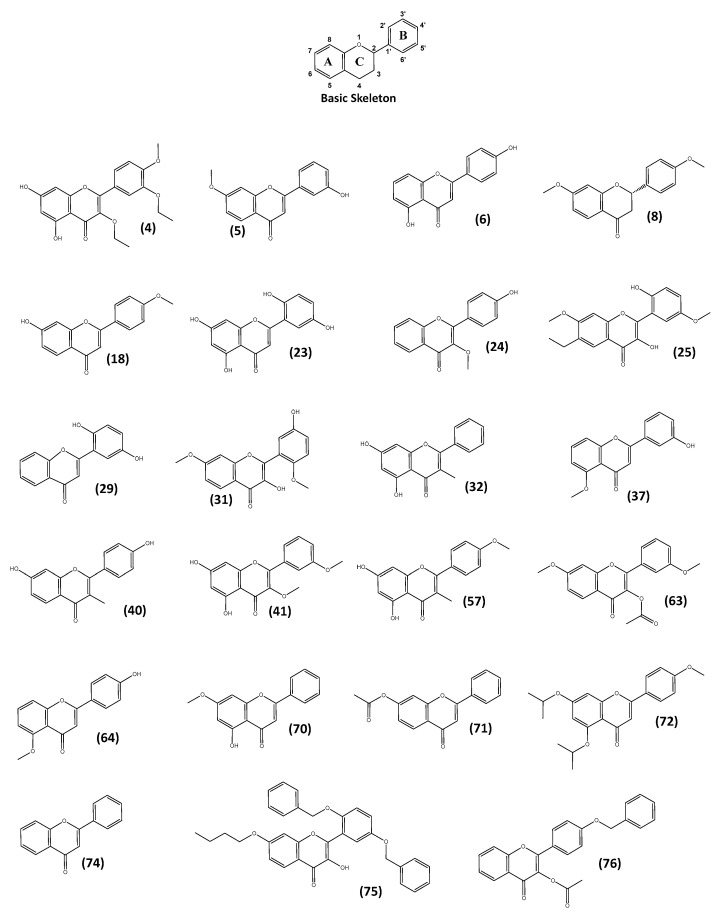
Chemical structures of 23 flavonoids with high neuro-differentiating ability in the first screen used in this study.

Recent studies have shown flavonoid compounds can hinder cell necrosis by mitigating the cellular stress response [39,40]. The research reported in this study indicates that these compounds may present chemotypes for AD therapy. Flavonoids are a group of polyphenolic compounds widely found in fruits, vegetables, and other food crops as secondary metabolites possessing a C15 skeleton. Flavonoids generally accumulate in plant cells in the form of glycosides, and they contain three rings (C6-C3-C6) as their basic skeleton (labelled A, B, and C in Figure 1) [41].

### Why Focus on Flavonoids

Epidemiological and nutritional intervention studies on animals and humans showed that an antioxidant-rich diet incorporating fruits and vegetables might prevent neurodegeneration, brain ischemia, and cognitive and motor decline due to aging, and might also reverse the effects of aging on neuronal function and behaviour. These neuroprotective effects are mainly due to the presence of antioxidants, especially polyphenolics such as flavonoids [42,43]. Recent dietary studies on different age groups showed that the consumption of a flavonoid-rich diet improved memory, focus, and cognition regardless of age. This neuroprotective effect is associated with their antioxidant and anti-inflammatory properties and the activation of synaptogenesis and neurogenesis pathways [44,45,46,47,48]. However, an unfilled gap exists in the experimental evidence regarding whether flavonoids can cross the blood–brain barrier (BBB) [42]. This unfilled gap prevents the development of flavonoid-based therapeutics. 

Therefore, many studies have focused on finding ways to increase the BBB permeability of flavonoids. Several bioavailability studies have been carried out to determine the BBB permeability of different flavonoids. One study showed that quercetin could pass the BBB if dispensed with α-tocopherol (vitamin E) [45]. Naringenin, hesperidin, and tea flavanol showed good BBB permeability after oral administration to mice [46,47,48,49,50]. Recently, novel drug delivery systems have been used to enhance flavonoids’ bioavailability and to overcome their unfavourable physical and chemical properties, such as inclusion complexes [51], nanostructured carriers [52], and solid lipid nanoparticles (SLN) [53]. Among them, the SLN drug delivery system has been used to enhance flavonoids’ delivery to the brain and hence increase their use in clinical settings [53].

Previous studies have also demonstrated that natural and synthetically produced flavonoid analogues have neuroprotective effects [54] and AchE inhibitory [55], GSK- and tau-aggregation inhibitory, and Aβ fibril formation inhibitory activities [56,57]. 

The principal constituent of green tea, epigallocatechin-3-gallate (EGCG), has been shown to mitigate Aβ-induced neurotoxicity by inhibiting BACE1 [58,59] and preventing oxidative stress and mitochondrial dysfunction by inhibiting NADPH oxidase activity [60]. It has also been found that flavonoid derivatives exhibit antiamnesic and vital memory functions in different experimental models of amnesia, along with inhibiting progressive neurodegeneration in AD [61]. 

Isoflavones isolated from soy have been shown to lessen oxidative stress and improve conditions related to ageing [62]. Hibifolin, as an example of a flavonol glycoside, hinders Aβ-induced neurotoxicity in cultured cortical neurons by decreasing caspase activation [63]. Icariin showed neuroprotective effects by inhibiting tau protein hyperphosphorylation [64]. Kaempferol has displayed protective effects against oxidative stress-induced cytotoxicity and reversed Aβ-induced impaired performance in a Y-maze test [65]. The protective role of quercetin in scopolamine-induced memory deficits has also been reported [62]. Nobiletin Is a citrus flavonoid that exhibits anti-neuroinflammatory effects by preventing the inflammatory response [66]. Also, recent studies have shown that flavonoids effectively block age-associated toxicity involved in AD pathways [67,68,69,70]. These findings have demonstrated that these flavonoids can cross the BBB to exert their effects. 

In vivo studies have shown that 7,8-dihydroxyflavone has neurotrophic activity in mice, which is mediated by its acting as a bioactive tyrosine kinase receptor B (TrKB) agonist [71].

The current study investigated the neurotrophic activity of 80 flavonoid compounds using a high-content screening approach to identify candidates that triggered neural differentiation and/or exhibited protective or synaptogenic activity. The neurite lengths and numbers, as well as the fluorescence intensity of synaptophysin and PSD95 were determined for compounds categorised as having high neuro-differentiating activity. Compounds exerting the highest neuro-differentiating and synaptogenic activity were then assayed for their neuroprotective potential. The structure–activity relationships of these compounds were then analysed to determine the structural properties that are fundamental in contributing to neuroprotective activity. Finally, a molecular docking study was carried out for the 3 lead flavonoid compounds and 21 proposed compounds, using the crystal protein structure of the tyrosine kinase B-d5 (TrKB-d5) domain (PDB code: 1HCF) to estimate the strength of binding between the tested compounds and this vital protein.

## 2. Results and Discussion

Based on the same C15 skeleton core structure, 80 structurally varied flavonoid compounds (Figure S1) were tested for their neurotrophic activity. DataWarrior software Version 5.5.0 [72] was used to calculate the chemical structure similarities between the molecules and to create a similarity tree using a Tanimoto approach (Figure S2). The flavone skeleton was identified as the starting marker, and the SkelSpheres descriptor was used to find the close similarities between the structures. SkelSpheres are the most accurate descriptors used to calculate the similarity between chemical graphs [72].

### 2.1. Screening of Neurotrophic Activity of the Compounds

To assess the potential neurotrophic activity of the compounds, the highest non-toxic dose of the compounds on SH-SH5Y cells was firstly evaluated using an MTT assay.

The compounds’ neuro-differentiating activity was then determined and was compared to that of the positive control, brain-derived neurotrophic factor (BDNF). The neurotrophic activity of the compounds was categorised as poor, medium, or high, as shown in Table S1. Of 80 flavonoids, 23 had high neuro-differentiating ability, 36 were poor, and 21 were ranked as medium neuro-differentiating ability (Table S1).

### 2.2. Validation of the Neurotrophic and Synaptogenic Activities of the Candidates

The neurite lengths and numbers, as well as the fluorescence intensity of synaptophysin and PSD95 were determined for 23 flavonoids categorised as having high neuro-differentiating ability in the first screen (Figure 1). Of the 23 flavonoids, 12 flavonoids (compounds (**4**), (**5**), (**23**), (**57**), (**63**), (**64**), (**70**), (**71**), (**72**), (**74**), (**75**), and (**76**)) showed neurotrophic activity as cells treated with these compounds had longer neurites and a higher number of neurites than the control. With regards to the synaptogenic activity, compounds (**4**), (**5**), (**6**), (**8**), (**18**), (**23**), (**24**), (**41**), and (**70**) induced the expression of synaptophysin while only compound (**4**) promoted the expression of PSD95. Taken together, compounds (**4**), (**5**), and (**23**) showed both neuro-differentiating and synaptogenic activity (in terms of the expression of synaptophysin).

### 2.3. Determination of the Neuroprotective Activity

The neuroprotective activity was assessed on lead compounds (**4**), (**5**), and (**23**), which showed both neuro-differentiating and synaptogenic activity (in terms of synaptophysin expression). Following differentiation of the cells with BDNF, the latter were pre-treated with the compounds for 24 h, after which the cells were treated with different insults. As such, cells were treated with 100 µm of H_2_O_2_, representing an oxidative stress model. Aβ 1–40 (35 µM) was used to represent the Alzheimer’s model, and glutamate (100 mM) represented the Parkinson’s model.

**Figure 2:**
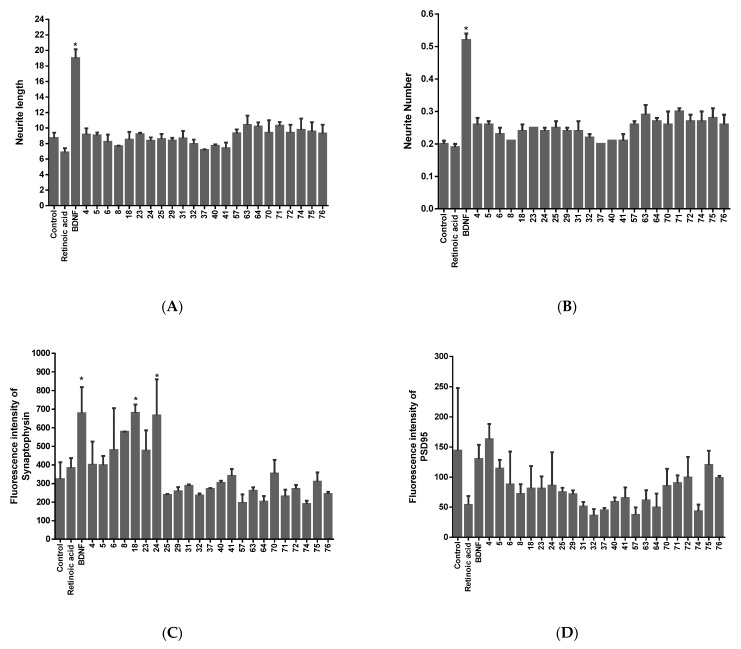
Neuro-differentiating activity of the compounds in terms of (**A**) neurite length, (**B**) neurite number, and synaptogenic activity in terms of (**C**) synaptophysin expression and (**D**) PSD95 expression. Data expressed as mean ± standard deviation. Significance was assessed using one-way ANOVA followed by Tukey post hoc; * *p* < 0.05 (vs. control) (BDNF: positive control, retinoic acid (RA): internal control, vehicle-treated: control).

**Figure 3:**
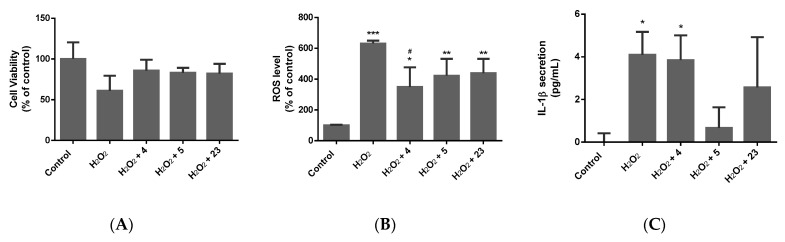
Effect of compounds (**4**), (**5**), and (**23**) on the oxidative stress model in terms of (**A**) cell viability, (**B**) ROS production, and (**C**) IL-1β secretion when exposed to H_2_O_2_. Data expressed as mean ± standard deviation. Significance was assessed using one-way ANOVA followed by Tukey post hoc; * *p* < 0.05, ** *p* < 0.01, *** *p* < 0.001 (vs. control); # *p* < 0.05 (vs. H_2_O_2_).

**Figure 4:**
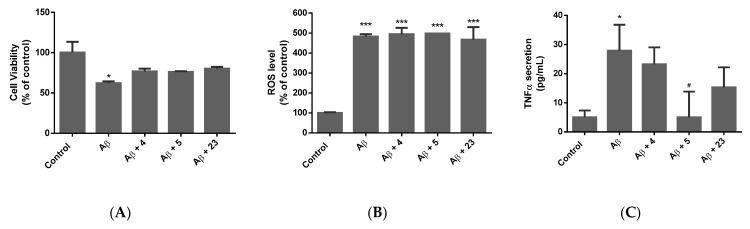
Effect of compounds (**4**), (**5**), and (**23**) on (**A**) cell viability, (**B**) ROS production, and (**C**) TNF-α secretion when exposed to Aβ 1–40. Data expressed as mean ± standard deviation. Significance was assessed using one-way ANOVA followed by Tukey post hoc; * *p* < 0.05, *** *p* < 0.001 (vs. control); # *p* < 0.05 (vs. Aβ 1–40).

**Figure 5:**
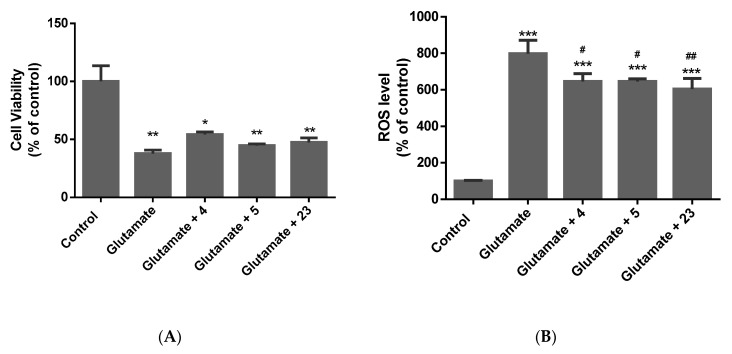
Effect of compounds (**4**), (**5**), and (**23**) on (**A**) cell viability and (**B**) ROS production when exposed to glutamate. Data expressed as mean ± standard deviation. Significance was assessed using one-way ANOVA followed by Tukey post hoc; * *p* < 0.05, ** *p* < 0.01, *** *p* < 0.001 (vs. control); # *p* < 0.05, ## *p* < 0.01 (vs. glutamate).

Following 72 h of incubation with the respective insult, cell viability, ROS production, neurite loss, and the secretion of pro-inflammatory cytokines, including TNFα and IL-1β, were measured (Figure 2, Figure 3, Figure 4 and Figure 5).

#### 2.3.1. Oxidative Stress Model

A 72 h incubation with 100 µM of H_2_O_2_ resulted in a 40% decrease in the cell number. Pretreatment with the compounds showed protective potential as cell death was inhibited by at least 20%. H_2_O_2_ promoted oxidative stress in differentiated SH-SY5Y cells, as evidenced by the increase, by at least 500%, of ROS production. Compounds (**4**), (**5**), and (**23**) decreased ROS production in the H_2_O_2_-treated cells, thus exerting antioxidant activity. The compounds also demonstrated anti-inflammatory activity as they counteracted the H_2_O_2_-induced increase in IL-1β secretion. The compounds, however, did not show any neuro-protective activity in this model, as they did not counteract the H_2_O_2_-induced loss in neurite number and length.

#### 2.3.2. Alzheimer’s Model

Following 72 h of incubation with Aβ 1–40, the SH-SY5Y cell number decreased by about 40%. Interestingly, the three compounds counteracted the Aβ 1–40-induced cell death. It is also noteworthy that the three compounds demonstrated anti-inflammatory activities by counteracting the over-secretion of TNF-α induced by Aβ 1–40. In this model, the three compounds did not show antioxidant activity, as no major alteration in the ROS level was observed.

#### 2.3.3. Parkinson’s Model

Glutamate showed a 60% decrease in cell number following 72 h of treatment. Compound (**4**) increased the cell number by at least 10%. Compounds (**4**), (**5**), and (**23**) exerted antioxidant activity by decreasing the ROS level. In this model, no major alteration in the secretion of pro-inflammatory cytokines was noted.

### 2.4. Structure–Activity Relationship (SAR) Analysis

#### 2.4.1. Structure–Activity Relationship (SAR) Analysis Compared with Neuro-Differentiating Activity

Upon closer observation of the bioactivity of the selected 80 flavonoids, some interesting trends can be highlighted that connect the exhibited neuro-differentiating activity with the structural features of 60 flavone compounds. The neuro-differentiating activity of the compounds was categorised as poor, medium, or high based on the morphology of the cells treated with the compound. The SAR determination was carried out for 60 flavones (to keep the primary scaffold identical for all flavone structures). Flavones are distinguished from other flavonoids by the presence of a double bond between C2-C3, oxidized at C4, and no substitution at the C3 position [41]. Fourteen compounds ((**4**), (**5**), (**6**), (**18**), (**23**), (**24**), (**29**), (**32**), (**37**), (**40**), (**41**), (**57**), (**64**), and (**70**)) showed high and 19 compounds ((**3**), (**9**), (**10**), (**11**), (**12**), (**14**), (**17**), (**19**), (**20**), (**26**), (**27**), (**30**), (**33**), (**36**), (**38**), (**39**), (**50**), (**55**), and (**61**)) had poor neuro-differentiating activity (Table S1). These flavonoid structures look very similar; however, the data demonstrate that the addition of hydroxy groups to the compound structures gives high neuro-differentiating activity, whereas compounds with few or very few hydroxy groups have poor activity. 

The solubility and relative total polar surface area of these compounds were calculated (Table S2 and Figure S3). The addition of two methoxy groups as well as hydroxy groups to the compounds enhanced their bioactivity.

The substitution pattern of hydroxy, methoxy, and substituent groups at C3, C5, and C7 on the chromene (i.e., oxygen-containing heterocyclic compounds) structure on ring A and at C3′, C4′, and C5′ on ring B of the structure gave high neuro-differentiating activity [73]. It has been reported previously that hydroxyl substitutions at C5, C7, and C3′ have neuroprotective effects against oxidative stress-induced neurotoxicity [74].

#### 2.4.2. Structure–Activity Relationship (SAR) Analysis Compared with Synaptogenic Activity

An SAR study was carried out (Tables S3–S5) for the 19 compounds ((**4**), (**5**), (**6**), (**18**), (**23**), (**24**), (**29**), (**32**), (**37**), (**40**), (**41**), (**57**), (**63**), (**64**), (**70**), (**71**), (**72**), (**74**), and (**76**)) showing neuro-differentiating and synaptogenic activities (Table S5). The data show that the number of hydroxyl or methoxy substitutions at the C5 and C7 positions of ring A and the C4′ position of ring B plays a vital role in determining the activity. As such, out of 19, 8 compounds ((**29**), (**37**), (**63**), (**64**), (**71**), (**72**), (**74**), and (**76**)) have lower synaptogenic activity due to the absence of hydroxyl substitutions at C5 and C7. Also, the data show that the presence of a methyl substitution group lowers the activity of compounds (**32**) and (**57**). Nine compounds ((**4**), (**5**), (**6**), (**18**), (**23**), (**24**), (**40**), (**41**), and (**70**)) show higher bioactivity compared to the others because of the presence of one or more hydroxyl or methoxy substitutions at the C5 and C7 positions of ring A and the C4′ position of ring B.

#### 2.4.3. Structure–Activity Relationship (SAR) Analysis Compared with Antioxidant Activity

Flavonoids can protect against damage from ROS [75,76,77]. The ROS scavenging properties of flavonoids make them effective anti-inflammatory and neuroprotective compounds [78,79]. A characteristic structural feature among flavonoid subclasses is the existence of a C_2_C_3_ double bond in conjugation with a position 4-carbonyl group in ring C, which plays a role in antioxidant activity [80]. Taken together, a combination of a 4-carbonyl group with a C_2_C_3_ double bond or other electron donating group efficiently delocalises the ring B electrons, thus significantly enhancing the compound’s antioxidant activity.

Substitution of hydroxyl groups at C5 and C7 on ring A and C3′ on ring B of the flavone structure plays a crucial role in antioxidant activity. The antioxidant activity of compounds (**4**), (**5**), and (**23**) can thus be attributed to the substitution at C5 and/or C7. It has been reported that flavones containing two hydroxyl substitutions in ring B and a hydroxyl substitution at C7 exhibit strong antioxidant properties [80]. Compound (**23**) has two hydroxyl groups on ring B and an -OH group at C7, and its antioxidant activity can be attributed to this structure. Also, it has been found that flavones containing hydroxyl groups at C3, C5, and C7 demonstrate a strong antioxidant effect [77] (Table S6), further supporting the antioxidant activity of compounds (**4**) and (**23**), which possess hydroxyl groups at C5 and C7.

#### 2.4.4. Structure–Activity Relationship (SAR) Analysis Compared with Anti-Inflammatory Activity

As compound (**5**) shows excellent anti-inflammatory activity compared to compounds (**4**) and (**23**), this indicates that the presence of a methoxy group at the C7 position in compound (**5**) enhances its anti-inflammatory activity. The presence of a hydroxyl group at C3′ may also enhance this activity (Table S7). It has been reported previously that the presence of a methoxy group at the C5 and C7 positions, and the presence of a hydroxyl group at the C3′ position enhance the anti-inflammatory activity of flavones [81], which matches our findings.

### 2.5. Molecular Docking Studies

#### 2.5.1. Molecular Docking Studies with the TRKB-d5 Domain (PDB: 1HCF)

**Figure 6:**
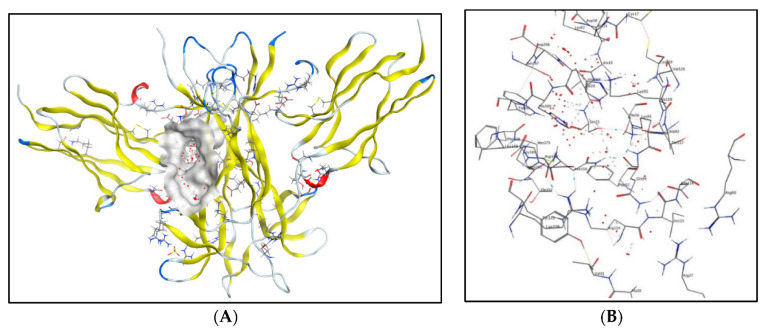
(**A**) PDB ID: 1HCF; Binding site in grey metallic colour; (**B**) Enlarged view of the binding site showing amino acid residues Trp^301^, Ser^21^, Met^379^, Cys^119^, Val^20^, Asn^350^, Ala^15^, Ala^18^, Ala^19^, His^299^, Phe^378^, Ala^118^, and Cys^17^ of the TrkB-d5 protein.

**Figure 7:**
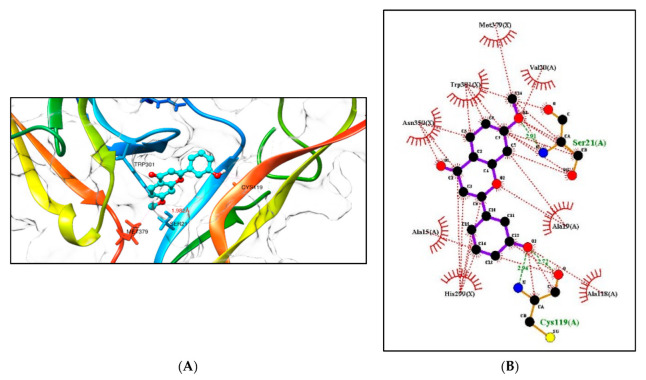
(**A**) Docking computations of the ligand structure (**5**) using Autodock Vina. The TRKB-d5 domain (ribbon structures) is coloured like a rainbow, and the aqua ball and stick frame structures denote the ligand structure. The dotted pink lines indicate hydrogen bond interactions between compounds and proteins. (**B**) LigPlot showing the strong hydrogen bonding of the substituents and the hydrophobicity at the C3′ and C7 positions with the nearest amino acids. Distance is in Å units.

Tropomyosin-related kinase B (TRKB) is a membrane-bound receptor that, upon the binding of BDNF, phosphorylates itself to initiate downstream signalling for neuronal survival and axonal growth. A molecular docking study was carried out for 3 lead flavonoid compounds (**4**), (**5**), and (**23**), as well as 21 hypothetical compounds with a flavonoid scaffold similar to compound (**5**), using the protein structure of the TRKB-d5 domain (PDB: 1HCF). The software Autodock Vina v. 1.2.0 (The Scripps Research Institute, La Jolla, CA, USA) [82] was used for docking, and the receptor site was found using MOE software 2022.02.09 [83], using the site finder for docking the flavonoid structures (Figure 6). Generally, the docking scores of ligands reflect their binding affinity to the target protein. It is interesting to note that lead compounds (**4**), (**5**), and (**23**) showed high binding affinity to the TRKB protein (involved in neurodifferentiation), which matches the in vitro results (Table 1). Docking of compound (**5**) at the active site of the TRKB protein and the types of interactions involved are shown in Figure 7.

**Figure 8:**
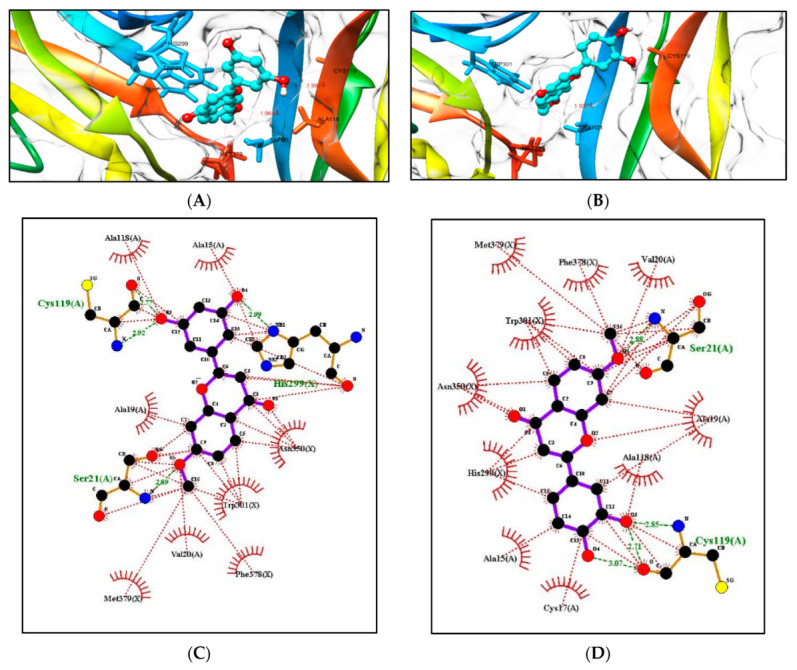
Docking computations of (**A**) structure (**5C**) and (**B**) structure (**5F**). The TRKB-d5 domain (ribbon structures) is coloured like a rainbow, and the aqua ball and stick frame structures denote the ligand structure. The dotted pink lines indicate hydrogen bond interactions between compounds and proteins. LigPlots of (**C**) structure (**5C**) and (**D**) structure (**5F**) showing strong hydrogen bonding of substituents and hydrophobicity at the C3′, C5′, and C7 positions with the nearest amino acids. Distance is in Å units.

Based on the structure of compound (**5**), seventeen hypothetical structures (**5A**–**5Q**) were docked in Autodock Vina using the same parameters. Clearly, the carbon positions 3′ and 5′ of ring B and the C-7 position of ring A play a vital role. Secondly, hydroxylation on ring B at C3′ and C5′ and methoxylation on ring A at C7 play a crucial role in bioactivity (Table 2). Hypothetical structures in blue (**5C**), (**5F**), (**5I**), and (**5L**) show the highest docking score, and these could be possible flavonoid structures for future neurodegenerative studies. Docking of hypothetical structures (**5C**) and (**5F**) at the active site of the TRKB protein and the types of interactions involved are shown in (Figure 8). The flavonoid structures from Table 2 with the highest docking score in blue ((**5C**), (**5F**), (**5I**), and (**5L**)) had the methoxy group at the C7 position of ring A replaced with a hydroxy group. Molecular docking was conducted in Autodock Vina by keeping the parameters the same (as described in the methods). This study shows that replacing a methoxy with a hydroxy group at position C7 has no effect on the binding affinity in the receptor pocket (Table 3). Hence, the presence of a methoxy or hydroxy group at position C7 plays a vital role.

#### 2.5.2. Additional Molecular Docking Studies with Human Phosphodiesterase 4B (PDB: 4MYQ) and AChE (PDB: 4BDT) and BuChE (PDB: 4BDS)

**Figure 9:**
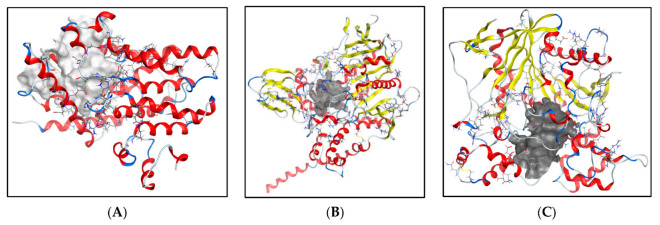
(**A**) Human phosphodiesterase 4B (PDB: 4MYQ), (**B**) AChE (PDB:4BDT), (**C**) BuChE (PDB:4BDS). Binding sites in grey metallic colour.

**Figure 10:**
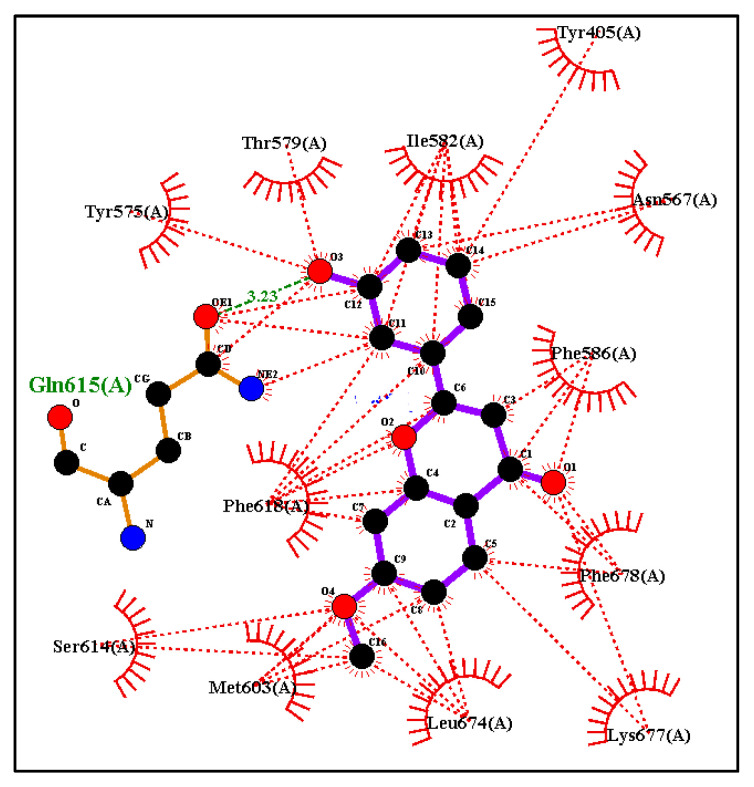
LigPlot of compound (**5**) showing strong hydrogen bonding of substituents and hydrophobicity at the C3′ and C7 positions with the nearest amino acids in the human phosphodiesterase 4B receptor. Distance is in Å units.

As discussed earlier, phosphodiesterase 4B is an important therapeutic target that may decrease the neuroinflammation in AD, and cholinesterase enzymes such as acetylcholinesterase (AChE) and butyrylcholinesterase (BuChE) have vital roles as co-regulators of the acetylcholine level. Therefore, additional molecular docking studies were carried out for the three lead flavonoid compounds (**4**), (**5**), and (**23**) using the protein structures of the human phosphodiesterase 4B domain (PDB:4MYQ) and the human X-ray structures of AChE (PDB:4BDT) and BuChE (PDB:4BDS) (Figure 9). The docking scores are shown in Table 4 and Table 5. The docking of compound (**5**) at the active site of the phosphodiesterase 4B receptor and the types of interactions involved are shown in Figure 10.

### 2.6. Pharmacophore Evaluation and ADMET Study

**Figure 11:**
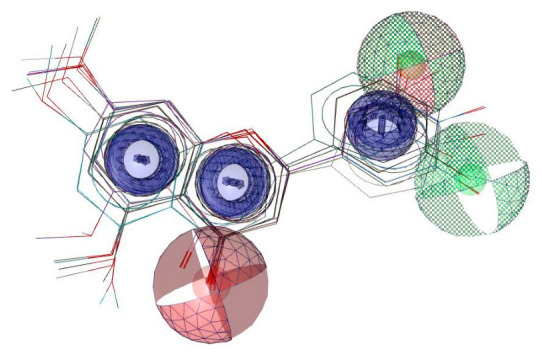
Common feature pharmacophore. Features are colour-coded with red, HBAs; green, HBDs; purple, ARs.

Using the pharmacophore ligand-based approach for these hypothetical compounds (Table 2) along with compound (**5**), a pharmacophore model was generated (Figure 11). The generated pharmacophore showed three main features: hydrogen bond acceptors (HBAs), hydrogen bond donors (HBDs), and aromatic rings (AR). The representative 3D and 2D pharmacophoric features of each structure are shown in Figure S4. Each compound constitutes individual pharmacophoric features, and from these individual characteristic pharmacophores, a merged pharmacophore with common features was generated, as shown in Figure 11. This common feature pharmacophore model with a score of 0.8979 showed certain features: two HBDs, two HBAs, and three ARs.

In silico evaluation of the ability of the top compounds (**4**), (**5**), and (**23**) to cross the blood–brain barrier was performed, and according to the results of the assessment, compound (**5**) was predicted to cross the BBB, while compounds (**4**) and (**23**) were predicted to be unable to cross the BBB.

## 3. Materials and Methods

### 3.1. Origin of Compounds

The flavonoid compounds (**1**) to (**80**) (donated by the late Professor R.H. Thomson) were obtained in purified form from the Marine Biodiscovery Centre Compounds Library, Department of Chemistry, University of Aberdeen, UK. They are as follows: 5,7-dimethoxy-4′-hydroxy flavonol (**1**), 5,7-dimethoxy-3,4′-dihydroxy flavanone (**2**), 4′-methoxy flavone (**3**), 5,7-dihydroxy-3,3′-diethoxy-4′-methoxy flavone (**4**), 3′-hydroxy-7-methoxyflavone (**5**), 5,4′-dihydroxyflavone (**6**), 7,2′-dimethoxyflavone (**7**), 7,4′-dimethoxy flavanone (**8**), 3-methyl-7,4′-diacetoxy flavone (**9**), 3′-hydroxy flavone (**10**), 7,3′,4′-trimethoxy flavone (**11**), 8-methoxy flavone (**12**), 7-methoxy flavanone (**13**), 7-methoxy flavone (**14**), 5,7-dimethoxy isoflavone (**15**), 7-hydroxy-5-methoxyflavone (**16**), 5,7,3′-triacetoxy-4′-methoxy flavone (**17**), 7-hydroxy-4′-methoxyflavone (**18**), 7-acetoxy-3-methyl-4′-methoxy flavone (**19**), 5-acetoxy flavone (**20**), 7-methoxy-5,4′-dihydroxy flavonol (**21**), 2′-hydroxyflavone (**22**), 5,7,2′,5′-tetrahydroxy flavone (**23**), 4′-hydroxy-3-methoxy flavone (**24**), 7,5′-dimethoxy-6-ethyl-2′-hydroxy flavonol (**25**), 5,7-dimethoxy-2′-hydroxyflavone (**26**), 2′-methoxy flavone (**27**), 6,8,4′-triacetoxy flavanone (**28**), 2′,5′-dihydroxy flavone (**29**), 7-methoxy-2′,5′-dihydroxyflavone (**30**), 7,2′-dimethoxy-5′-hydroxy-flavonol (**31**), 5,7-dihydroxy-3-methyl-flavone (**32**), 3′,4′-dimethoxy flavone (**33**), 5,7-dihydroxy-3-ethoxy-2′-methoxy-flavone (**34**), 5,7-dimethoxy-4′-alloxy-flavanone (**5.35**), 7-acetoxy-3-methyl flavone (**36**), 5-methoxy-3′-hydroxy-flavone (**37**), 5,7,4′-tri-isopropoxy-3′-methoxy flavone (**38**), 5,7,3′-tri-propoxy-4′-methoxy flavone (**39**), 7,4′-dihydroxy-3-methyl flavone (**40**), 5,7-dihydroxy-3,3′-dimethoxy flavone (**41**), 7,2′-dimethoxy-5′-hydroxy-flavone (**42**), 7,3′-dimethoxy flavonol (**43**), 7,4′-dimethoxy-5-hydroxy-flavonol (**44**), 5,7,4′-trihydroxy-3-methyl flavone (**45**), 3′-methoxy flavone (**46**), 3-methoxy-4′-benzoxy flavone (**47**), 5,7-dimethoxy-3′-flavone (**48**), 7-methoxy-4′-benzoxy-flavonol (**49**), 6,8-dimethoxy flavone (**50**), 5-hydroxy-3,7,4′-trimethoxy flavone (**51**), 5-(5,7-dimethoxy-4-oxo-4H-chromen-2-yl)-2-hydroxybenzaldehyde (**52**), 5,2′-dihydroxy-7-methoxyflavone (**53**), 4′-benzoxy-flavonol (**54**), 4′-benzoxy flavone (**55**), 3-methoxy-3′-hydroxy-flavone (**56**), 3-methyl-5,7-dihydroxy-4′-methoxyflavone (**57**), 7-hydroxy flavonol (**58**), 7-methoxy-3,5,4′-triacetoxy flavone (**59**), 5-hydroxy-3-ethoxy-7,4′-dimethoxy flavone (**60**), 3,7,3′-trimethoxy flavone (**61**), 3-methoxy-5-hydroxy-7-benzoxy flavone (**62**), 3-acetoxy-7,3′-dimethoxy flavone (**63**), 5-methoxy-4′-hydroxy flavone (**64**), 3-methoxy-4′-acetoxy flavone (**65**), 5,7,4′-trimethoxy flavonol (**66**), 5-hydroxy-3,7,4′-trimethoxy-3′ethoxy flavone (**67**), 5-(5,7-dimethoxy-4-oxo-4H-chromen-2-yl)-2-methoxybenzaldehyde (**68**), 3,5-dimethoxy-7-hydroxyflavone (**69**), 5-hydroxy-7-methoxyflavone (**70**), 7-acetoxyflavone (**71**), 5,7-diisopropoxy-4′-methoxy flavone (**72**), 7-isopropoxy-4′-methoxyflavone (**73**), flavone (**74**), 7-butoxy-2′,5′-dibenzoxyflavonol (**75**), 3-acetoxy-4′-benzoxy flavone (**76**), 7,8,5′-dimethoxy-6-ethyl-2′-hydroxy flavonol (**77**), 3-methyl-7-hydroxy-4′-methoxy flavone (**78**), 5-acetoxy-7,2′-dimethoxy flavone (**79**), and 7-isopropoxyflavone (**80**).

### 3.2. Determination of the Highest Non-Toxic Dose of Compounds (Method S4.2)

SH-SY5Y cells were passaged and seeded at a density of 10,000 cells/well. The next day, the cells were treated with 200 µL of 100 pM to 10 µM of compounds for 48 h. After the treatment time, the supernatant was removed, and 100 µL of 0.5 mg/mL of MTT dye was added for 2 h. The MTT dye was removed, and 50 µL of DMSO was then added.

The absorbance was measured at 450 nm. The percentage cell viability was calculated as per the equation below:

$$\mathrm{Cell}\;\mathrm{viability}\;\left(\%\right)=\frac{\mathrm{Absorbance}\;\mathrm{of}\;\mathrm{extract}\;\mathrm{treated}\;\mathrm{cells}\;-\;\mathrm{Absorbance}\;\mathrm{of}\;\mathrm{Blank}}{\mathrm{Absorbance}\;\mathrm{of}\;\mathrm{control}\;\mathrm{cells}\;-\;\mathrm{Absorbance}\;\mathrm{of}\;\mathrm{Blank}}$$

A dose-response curve was generated for each compound, and the highest non-cytotoxic concentration was determined based on the selection criterion of 95 ± 5% of cell viability.

### 3.3. Screening of Neurotrophic Activity of the Compounds (Method S4.3)

**Figure 12:**
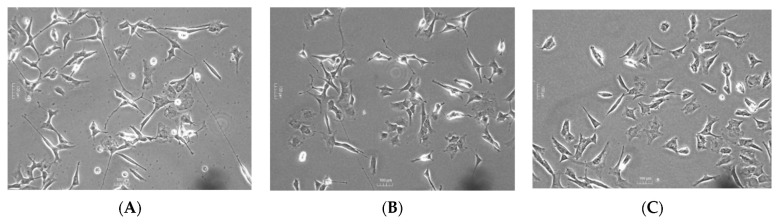
Representative figures of SH-SY5Y cells treated with the different compounds with (**A**) high, (**B**) medium, or (**C**) poor differentiation abilities.

SH-SY5Y cells were passaged and seeded at a confluency of 7000 cells/well in 24-well TPP plates. Following 24 h of plating, cells were treated with 10 µM of retinoic acid (RA) in 10% FBS medium for 3 days. RA-treated cells were then incubated with 500 µL of the highest non-cytotoxic concentration of the compounds plus 50 ng/mL of BDNF or vehicle in 1% FBS medium for an additional 3 days. The differentiation potentials of the 80 compounds were compared to that of BDNF, and they were categorized as poor, medium, or high, as illustrated in Figure 12.

### 3.4. Assessment of the Neurotrophic and Synaptogenic Activities of the Candidates by High Content Screening (HCS) (Method S4.4)

SH-SY5Y cells were passaged and seeded at a confluency of 3000 cells/well (100 µL) in 96-well TPP plates. Following 24 h of plating, cells were treated with 10 µM of RA (100 µL) in 10% FBS medium for 3 days. RA-treated cells were then incubated with 100 µL of the highest non-cytotoxic concentration of the compounds plus 50 ng/mL of BDNF or vehicle in 1% FBS medium for an additional 3 days. Following the incubation period, BDNF-treated cells were refreshed with 50 ng/mL BDNF for an additional 5 days.

After the incubation period, cells were washed and fixed with 3.7% formaldehyde. Following permeabilization, the cells were blocked and then incubated overnight with the primary antibodies (rabbit anti-TUBB3 (1:500), chicken anti-MAP2 (1:5000), rabbit anti-synaptophysin (1:200), and mouse anti-PSD95 (1:200)). The next day, the cells were washed and incubated with 1 µg/mL Hoechst and the respective secondary antibody (donkey anti-rabbit Alexa Fluor 488, goat anti-chicken Alexa Fluor 594, and goat anti-mouse Alexa Fluor 750 (1:1000)) for 1 h. Images of the fluorescently-tagged cells were captured and analysed by the HCS CX5 to determine the neurite length and neurite number as well as the fluorescence intensity of synaptophysin and PSD95.

### 3.5. Compound and Insult Treatment on Differentiated SH-SY5Y Cells (Method S4.5)

SH-SY5Y cells were passaged and seeded at a confluency of 3000 cells/well (100 µL) in 96-well TPP plates. Following 24 h of plating, cells were treated with 10 µM of RA (100 µL) in 10% FBS medium for 3 days. Cells were then incubated with 50 ng/mL of BDNF in 1% FBS medium for an additional 3 days. Following the incubation period, BDNF-treated cells were refreshed with 50 ng/mL BDNF for an additional 5 days.

After the differentiation of the SH-SY5Y cells, the latter were pre-treated with the highest non-toxic dose of the compounds for 24 h. The cells were then exposed to 35 µM of Aβ 1–40, 100 mM of glutamate, or 100 µM of H_2_O_2_. After 72 h, cell viability, ROS production, and secretion of pro-inflammatory cytokines were assessed.

#### 3.5.1. Assessment of Cell Viability and ROS Production (Method S4.5.1)

After 72 h, the cells were stained with 1 µg/mL of Hoechst 33,342 (nuclear staining), 1 µg/mL of propidium iodide (staining of dead cells), and 5 µM of CellROX™ Deep Red Reagent (ROS marker) which purchased from Life Technologies Corporation, Eugene, OR, USA. The plate was then read using the HCS CX5 at a magnification of 10x. The number of nuclei of live and dead cells, as well as the fluorescence intensity of the CellROX™ Deep Red Reagent, were determined by the HCS CX5.

#### 3.5.2. Investigation of Secretion of TNFα and IL-1β (Method S4.5.3)

The supernatant was collected and ELISA was carried out as per the manufacturer’s protocol. A Greiner plate was coated with 100 µL of either TNFα (DY210-05) or IL-1β (DY201-05) capture antibody overnight. Following washing, the wells were blocked and incubated with 100 µL of the sample or standard. The detection antibody was added for 2 h. Following washing, streptavidin-HRP was added. TMB solution was then added to each well for 20 min, after which 2 N HCl was added to stop the reaction. The absorbance was measured at 450 nm, and the levels of TNFα and IL-1β were calculated following plotting of the standard curves.

### 3.6. Data Analysis and Visualisation

Structure similarity charts showing the structure similarity of the AQs using SkelSpheres as molecular descriptors were generated using DataWarrior software Version 5.5.0 [72]. The structure–activity relationship of the flavonoids was analysed using the SAR function in DataWarrior software Version 5.5.0 that helped analyse the different substituents at the different positions of the 80 flavonoids.

### 3.7. Molecular Docking Experiments

The receptor site was predicted using the MOE Site Finder program [83], which uses a geometric approach to calculate putative binding sites in a protein. Molecular docking analysis was performed using Autodock Vina v. 1.2.0 (The Scripps Research Institute, La Jolla, CA, USA) docking software [82]. The protein structure of the TRKB-d5 domain (PDB:1HCF) [84] was visualised to perform docking computation for compounds (**4**), (**5**), (**23**), and 21 imaginary compounds.

The box centre and size coordinates were 7.07373 × 4.25074 × 19.0761 and 14.8679 × 27.1588 × 35.4203 around the active site. All coordinates used Angstrom units. Default search parameters were used where the number of binding modes was 10, exhaustiveness was 8, and the maximum energy difference was 3 kcal/mol. Chimera 1.16 [85], Pymol2 [86], and LigPlot^+^ software Version 2.2 [87] were used for visualization and calculation of protein–ligand interactions. Additional Molecular Docking Studies with human phosphodiesterase 4B (PDB: 4MYQ) [88], AchE (PDB:4BDT) [89], and BuChE (PDB:4BDS) [89] were also performed in a similar manner as discussed above.

### 3.8. 3D Pharmacophore Model Generation and ADMET Study

LigandScout 4.4.8 (Inte: Ligand) Advanced software [90,91] (evaluation license key: 81809629175371877209) was used to generate a 3D pharmacophore model. The espresso algorithm was used to create a ligand-based pharmacophore. The generated pharmacophore model compatibility with the pharmacophore hypothesis was created using the default settings for LigandScout. The best model was selected from the 10 generated models.

The SwissADME online server [92] was used to predict the BBB permeation of the promising flavonoids using their SMILES strings.

## 4. Conclusions

In this study, 80 structurally varied flavonoid compounds were tested for their neuroprotective activity. Compounds (**4**), (**5**), and (**23**) were the lead compounds based on their neurotrophic and synaptogenic activities. Compound (**5**) showed the top-performing neuroprotective activity from among these three. 

Based on these bioactivity results, a SAR study was carried out, showing that hydroxy and methoxy substituent groups in the scaffold play a major role in the activity. Substitution of hydroxy groups in rings A and B shows higher neurodifferentiation activity. It was also clear that positions C3′ and C5′ of ring B and C5, as well as the C7 position of ring A, play vital roles in the antioxidant and anti-inflammatory activities. 

Based on the SAR results, a major molecular docking study was conducted for the three lead flavonoid compounds (**4**), (**5**), and (**23**), as well as twenty-one hypothetical compounds having a flavonoid scaffold similar to compound (**5**), using the protein structure of the TRKB-d5 domain. Compounds (**4**), (**5**), and (**23**) showed high binding affinity to the TrkB-d5 protein, matching the cellular in vitro results of neurotrophic activity as well as the SAR results. Molecular docking of the 21 hypothetical compounds clearly indicates that the presence of a hydroxy substituent group at position C3′ as well as at C5′ of ring B and a methoxy or hydroxy substituent group at the C7 position of ring A play a vital role in neuroprotective, antioxidant, and anti-inflammatory activity. This study clearly shows that these flavonoid structures could play a vital role in neurodegenerative research. 

Additionally, molecular docking studies with human phosphodiesterase 4B, AChE, and BuChE also support the proposal that the top candidate compounds could benefit future neurodegenerative disease treatment. These results led to the proposal of a pharmacophore model to help guide future studies.

## Figures and Tables

**Table 1:** The docking scores of the lead flavones (**4**), (**5**), and (**23**) with the TRKB-d5 domain (PDB: 1HCF).

Compound	Ring A		Ring B				Docking Score (kcal/mol)
	C5	C7	C2′	C3′	C4′	C5′	
**5**		OCH_3_		OH			**−9.5**
**4**	OH	OH			OCH_3_	OCH_2_CH_3_	−8.4
**23**	OH	OH	OH			OH	−9.4

**Table 2:** Docking scores of hypothetical structures with the TRKB-d5 protein; compound (**5**) is highlighted in green (as the standard).

Compound	Ring A		Ring B				Docking Score (kcal/mol)
	C5	C7	C2′	C3′	C4′	C5′	
**5**		OCH_3_		OH			−9.5
**5A**	OCH_3_	OCH_3_		OH			−8.5
**5B**	OCH_3_			OH			−8.8
**5C**		OCH_3_		OH		OH	−9.7
**5D**	OCH_3_	OCH_3_		OH		OH	−8.7
**5E**	OCH_3_			OH		OH	−8.9
**5F**		OCH_3_				OH	−9.4
**5G**	OCH_3_	OCH_3_				OH	−8.3
**5H**	OCH_3_					OH	−8.7
**5I**		OCH_3_		OH	OH		−9.6
**5J**	OCH_3_	OCH_3_		OH	OH		−8.6
**5K**	OCH_3_			OH	OH		−9
**5L**		OCH_3_			OH	OH	−9.5
**5M**	OCH_3_	OCH_3_			OH	OH	−8.6
**5N**	OCH_3_				OH	OH	−9
**5O**		OCH_3_		OH	OH	OH	−8.9
**5P**	OCH_3_	OCH_3_		OH	OH	OH	−8.4
**5Q**	OCH_3_			OH	OH	OH	−8.2

**Table 3:** Replacing the methoxy group with the hydroxy group has no effect on binding affinity.

Docking Score (with -OCH_3_) on Ring A (kcal/mol)	Docking Score (Replacing -OCH_3_ with -OH) on Ring A (kcal/mol)
−9.7	−9.6
−9.4	−9.3
−9.6	−9.5
−9.5	−9.5

**Table 4:** The docking scores of the lead flavones (**4**), (**5**), and (**23**) with the *phosphodiesterase 4B* receptor (PDB:4MYQ).

Compound	Ring A		Ring B				Docking Score (kcal/mol)
	C5	C7	C2′	C3′	C4′	C5′	
**5**		OCH_3_		OH			−10.2
**4**	OH	OH			OCH_3_	OCH_2_CH_3_	−8.9
**23**	OH	OH	OH			OH	−10.0

**Table 5:** The docking scores of the lead flavones (**4**), (**5**), and **(23**) with AChE (PDB:4BDT) and BuChE (PDB:4BDS).

Compound	Ring A		Ring B				Docking Score (kcal/mol) PDB:4BDT	Docking Score (kcal/mol) PDB:4BDS
	C5	C7	C2′	C3′	C4′	C5′		
**5**		OCH_3_		OH			−9.5	−9.3
**4**	OH	OH			OCH_3_	OCH_2_CH_3_	−8.0	−8.5
**23**	OH	OH	OH			OH	−9.8	−9.1

## Data Availability

The raw data supporting the conclusions of this article will be made available by the authors, without undue reservation.

## Supplementary Materials

The following supporting information can be downloaded at: https://www.mdpi.com/article/10.3390/molecules28207188/s1. Figure S1. Chemical structures of flavonoids used in this study. Figure S2. Structure similarity of the most structurally related flavonoids in the present study using DataWarrior software, Version 5.5.0. Figure S3. ClogP values of compounds from Table S2. Figure S4. 3D and 2D representations of pharmacophoric features of the hypothetical compounds (**5A**–**5Q**) used in 3D pharmacophore generation. Table S1. Values of the highest non-cytotoxic dose of the 80 flavonoids. The neuro-differentiating activity of the compounds was categorised as poor, medium, or high (results provided by Axonova). Table S2. ClogP values were generated (using Ligand Scout software, Version 4.4.8) to check the solubility and TPSA values of the compounds in the SAR study. Table S3. The presence of more hydroxyl groups in the 14 flavonoid structures increases their neuro-differentiating activity. Table S4. Fewer hydroxyl groups in the 19 flavonoid structures gives poor neuro-differentiating activity. Table S5. The presence of one or more than one hydroxyl or methoxy substitution at the C5 and C7 position of ring A and the C4′ position of ring B of nine flavonoids shows higher activity. The absence of hydroxyl groups at the C5 and C7 position of eight compounds shows lower activity. Table S6. Flavonoids **(4**), (**5**), and (**23**) show antioxidant activity. Position C5, C7, C3′, and C4′ hydroxylation exhibits solid antioxidant properties. Table S7. Compound (**5**) shows excellent anti-inflammatory activity compared to the other two compounds (**4**) and (**23**) because of 3′-hydroxylation taking place along with 7-methoxylation greatly enhances its anti-inflammatory properties. (Method S4.2) Protocol for determining the highest non-cytotoxic dose of the compounds by the MTT assay (provided by Axonova). (Method S4.3) Protocol for shortlisting the candidate with neurotrophic activity (provided by Axonova). (Method S4.4) Protocol for assessing the neurotrophic and synaptogenic activities of the candidates by HCS (provided by Axonova). (Method S4.5) Compound and insult treatment of differentiated SH-SY5Y cells (provided by Axonova). (Method S4.5.1) Assessment of cell viability and ROS production (provided by Axonova). (Method S4.5.2) Determination of neurite loss (provided by Axonova). (Method S4.5.3) Investigation of the secretion of TNFα and IL-1β (provided by Axonova).

## Sample Availability

Samples of the compounds are available from the authors.
