# Supplementary material for: Anti-Neurodegenerating Activity: Structure–Activity Relationship Analysis of Flavonoids
Source: Molecules. 2023 Oct 20;28(20):7188. doi: 10.3390/molecules28207188 (PMC10609304; doi:10.3390/molecules28207188)
Supplement: Supplementary file 1 [file molecules-28-07188-s001.zip › molecules-2665083-supplementary.pdf]

Supplementary Information

Anti-Neurodegenerating Activity: Structure–Activity Relationship Analysis of Flavonoids

Gagan Preet [1,†], Ahlam Haj Hasan [1,2,†], Piteesha Ramlagan [3], Shameem Fawdar [3], Fabien Boulle [3] and Marcel Jaspars [1,*]

[1] Marine Biodiscovery Centre, Department of Chemistry, University of Aberdeen, Aberdeen AB24 3UE, UK; gagan.preet1@abdn.ac.uk (G.P.); a.hajhasan.20@abdn.ac.uk (A.H.H.)

[2] Department of Medicinal Chemistry and Pharmacognosy, Faculty of Pharmacy, Jordan University of
Science and Technology, Irbid 22110, Jordan

[3] Axonova Ltd., Grand Port 51405, Mauritius; p.ramlagan@axonova-pharma.com (P.R.); s.fawdar@axonova-pharma.com (S.F.); f.boulle@axonova-pharma.com (F.B.)

* Correspondence: m.jaspars@abdn.ac.uk

† These authors contributed equally to this work.

List of contents

**Figure S1.** Chemical structures of Flavonoids used in this study.	3
**Figure S2.** Structure similarity of the most structurally related flavonoids in the present study using DataWarrior Software.	5
**Figure S3.** ClogP Values of compounds from table S2.	9
**Figure S4.** 3D and 2D representations of pharmacophoric features of the hypothetical compounds (5A – 5Q) used in 3D pharmacophore generation.	12
**Table S1.** Values the highest non-cytotoxic dose of the 80 flavonoids. The Neuro-differentiating activity of the compounds was categorised as poor, medium, or high. (Results provided by Axonova).	5
**Table S2** ClogP Values were generated (using Ligand Scout Software) to check the solubility and TPSA values of the compounds in the SAR study.	8
**Table S3.** The presence of more hydroxyl groups in the 14 flavonoid structure increases the neuro-differentiating activity.	9
**Table S4.** Fewer hydroxyl groups in the 19 flavonoid structures give poor neuro-differentiating activity.	10
**Table S5.** The presence of one or more than one hydroxyl or methoxy substitution at the C5 and C7 position of Ring A and C4' position of Ring B of nine flavonoids shows higher activity; the absence of hydroxyl groups at the C5 and C7 position of 8 compounds showing lower activity.	10
**Table S6.** Flavonoids **(4)**, **(5)** & **(23)** show antioxidant activity; position C5, C7, C3', and C4' hydroxylation exhibits solid antioxidant property.	11

**Table S7.** Compound **(5)** shows excellent anti-inflammatory activity compared to the other two compounds **(4) & (23)** because 3′-hydroxylation taking place along with 7-methoxylation greatly enhances anti-inflammatory properties.	12
**(Method S4.2)** Protocol for determining the highest non-cytotoxic dose of the compounds by the MTT assay *(Provided by Axonova).*	16
**(Method S4.3)** Protocol for shortlisting the hits candidate with neurotrophic activity *(Provided by Axonova).*	17
**(Method S4.4)** Protocol for assessing the neurotrophic and synaptogenic activities of the hit candidates by HCS *(Provided by Axonova).*	17
**(Method S4.5)** Compound and insult treatment on differentiated SH-SY5Y cells *(Provided by Axonova).*	18
**Method S4.5.1)** Assessment of cell viability and ROS production *(Provided by Axonova).*	19
**(Method S4.5.2)** Determination of neurite loss *(Provided by Axonova).*	19
**(Method S4.5.3)** Investigation of secretion of TNFα and IL-1β *(Provided by Axonova).*	19

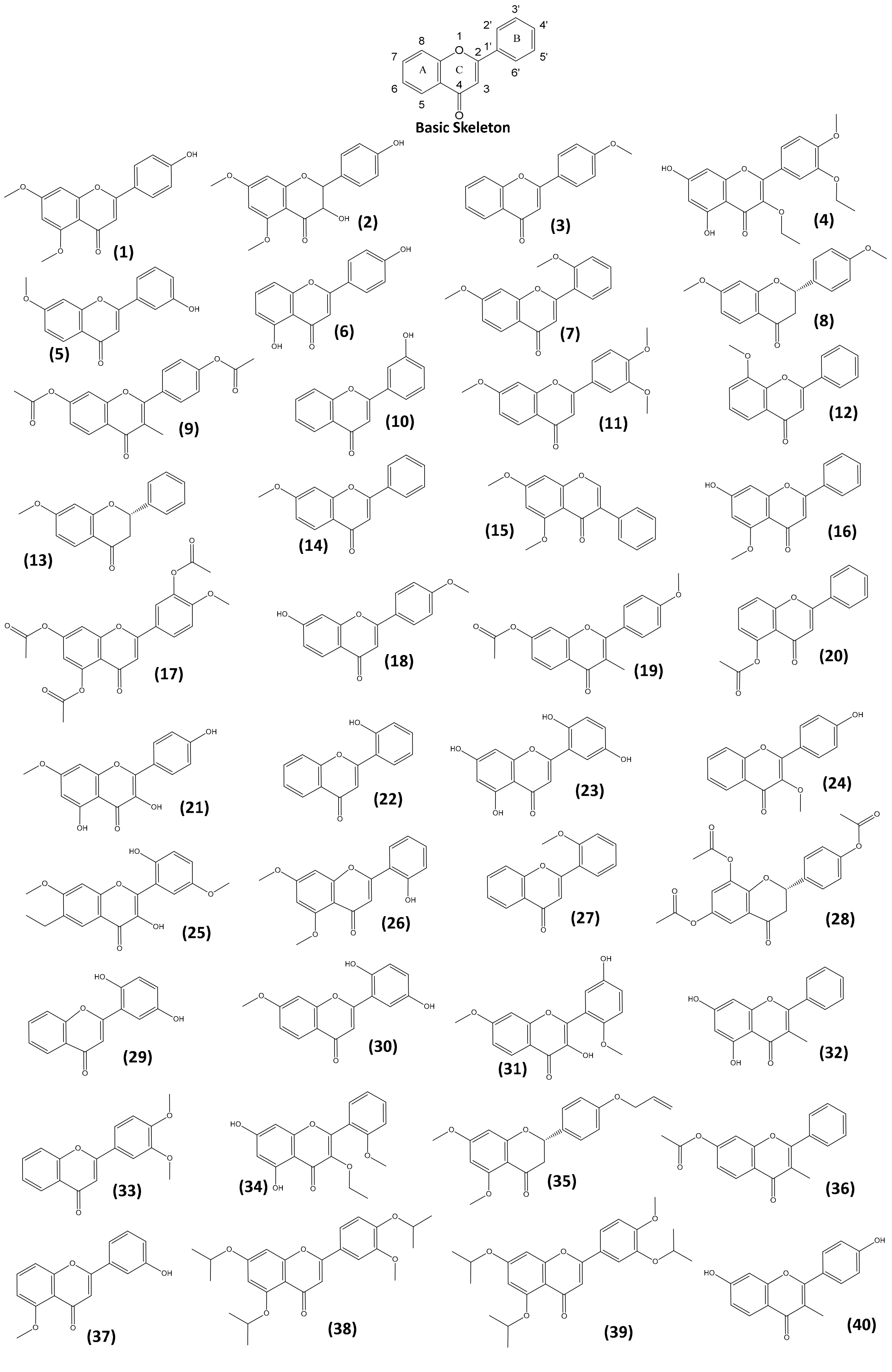
Basic Skeleton
A
B
C
(1)
(2)
(3)
(4)
(5)
(6)
(7)
(8)
(9)
(10)
(11)
(12)
(13)
(14)
(15)
(16)
(17)
(18)
(19)
(20)
(21)
(22)
(23)
(24)
(25)
(26)
(27)
(28)
(29)
(30)
(31)
(32)
(33)
(34)
(35)
(36)
(37)
(38)
(39)
(40)

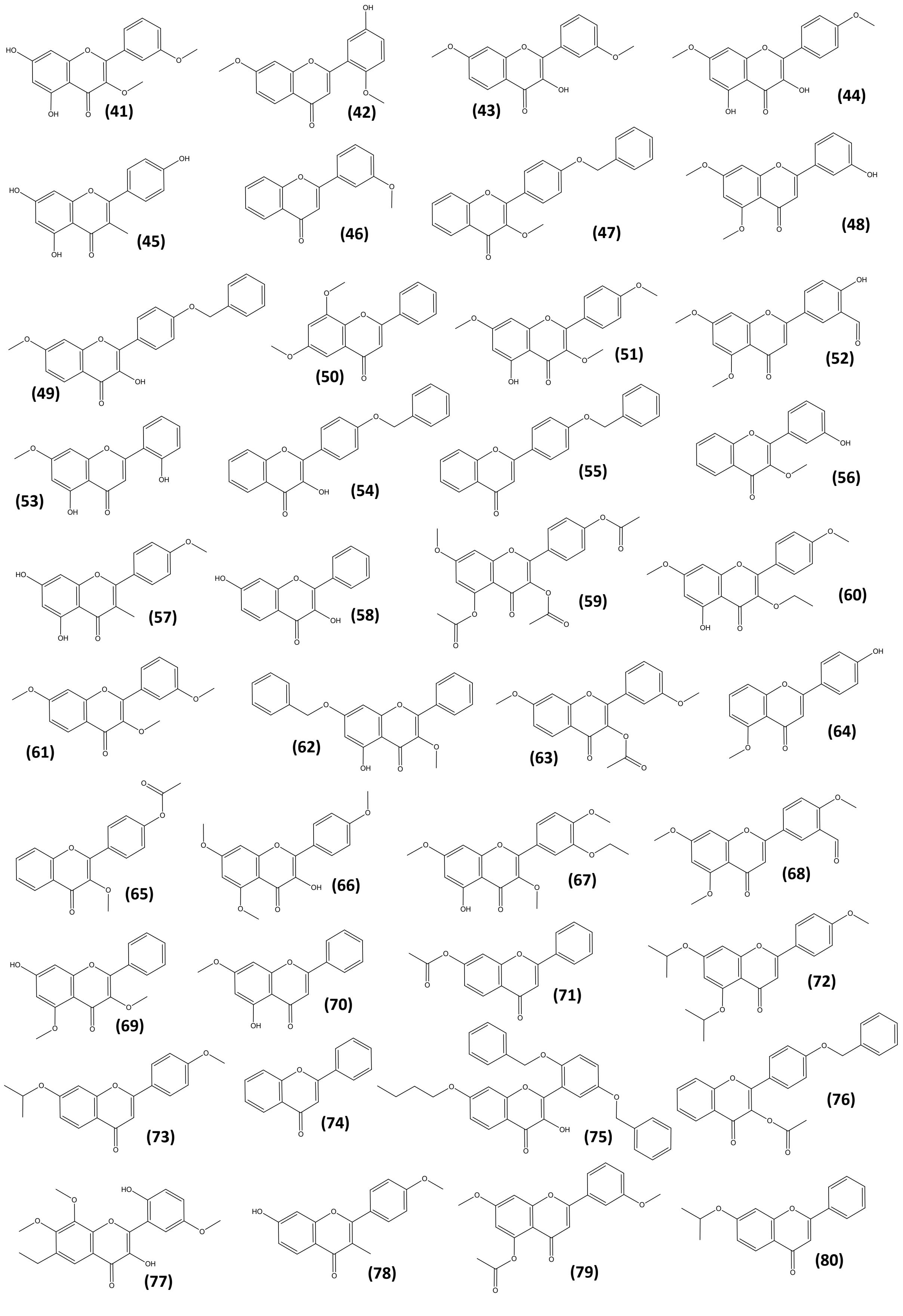

Figure S1. Chemical structures of Flavonoids used in this study.

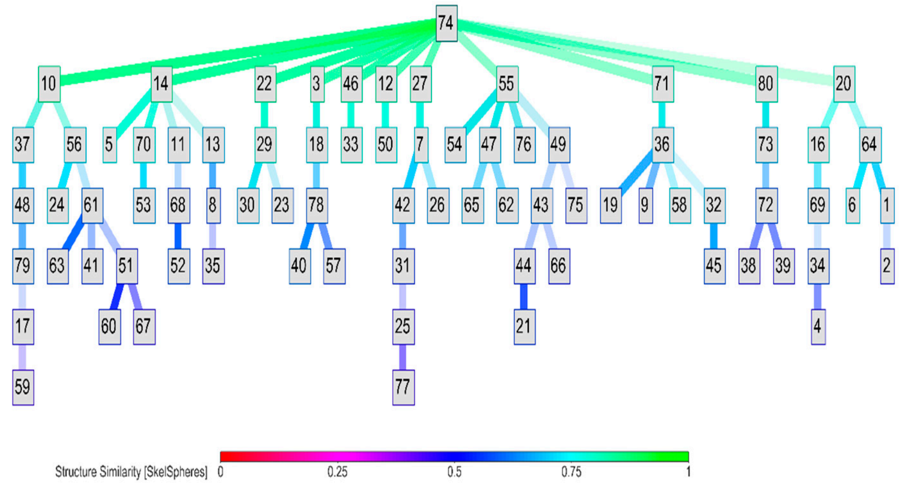

Figure. S2 Structure similarity of the most structurally related flavonoids in the present study using DataWarrior Software [71]. DataWarrior software calculates a similarity value between the molecules in a two-step process. Firstly, certain molecular features are extracted by processing the molecular graphs of the dataset consisting all molecules. These are gathered into an abstract molecule description called a descriptor. In simple terms, the descriptor comprises of a binary array of which every bit express whether a specific feature is situated in the molecule. These binary descriptors can also be termed as fingerprints. In the second step, the descriptors of the molecules are compared with some logic to establish the true similarity value, which indicates how much the two compounds have in common. In the case of binary descriptor, DataWarrior divides the total number of common features by the number of features available in any of the two molecules. This is referred to as Tanimoto similarity. The structural similarity was identified using Datawarrior software, where the flavone skeleton was identified as starting marker, and Skelspheres was used as a molecular descriptor to find the close similarity between the structures. Skelspheres descriptors are used to achieve the best clustering or similarity analysis results. When a more fine structural similarity is required, the SkeletonSpheres descriptor should be adopted. It is associated to the SphereFp, but also recognise stereochemistry, counts duplicate frag-ments, conceals hetero-atom reduced skeletons, and has two times the resolution lead-ing to lesser hash collisions. It is the most authentic descriptor for calculating the simi-larities of chemical graphs. [71]

Table S1. Values the highest non-cytotoxic dose of the 80 flavonoids. The Neuro-differentiating activity of the compounds was categorised as poor, medium, or high. (Results provided by Axonova)

S.No.	Compound	Highest non-toxic dose	Neuro-differentiating ability
1	1	1 nM	Poor
2	2	10 nM	Poor
3	3	1 µM	Poor
4	4	1 µM	High
5	5	1 nM	High
6	6	1 µM	High
7	7	100 pM	Medium
8	8	1 µM	High
9	9	1 nM	Poor
10	10	Toxic at 100 pM	Poor

11	11	10 nM	Poor
12	12	Toxic at 100 pM	Poor
13	13	1 µM	Poor
14	14	1 µM	Poor
15	15	100 nM	Poor
16	16	100 nM	Poor
17	17	1 nM	Poor
18	18	1 nM	High
19	19	10 µM	Poor
20	20	100 pM	Poor
21	21	100 pM	Medium
22	22	1 nM	Medium
23	23	1 µM	High
24	24	1 nM	High
25	25	1 nM	High
26	26	1 µM	Poor
27	27	10 µM	Poor
28	28	1 µM	Poor
29	29	10 nM	High
30	30	1 µM	Poor
31	31	1 µM	High
32	32	1 µM	High
33	33	10 µM	Poor
34	34	10 µM	Poor
35	35	1 µM	Poor
36	36	10 µM	Poor
37	37	10 nM	High
38	38	10 µM	Poor
39	39	10 µM	Poor
40	40	1 µM	High
41	41	10 nM	High
42	42	1 µM	Medium
43	43	1 µM	Medium
44	44	1 µM	Medium
45	45	1 nM	Poor
46	46	1 nM	Medium
47	47	10 nM	Medium

48	48	1 μM	Poor
49	49	1 nM	Poor
50	50	100 nM	Poor
51	51	1 nM	Poor
52	52	100 nM	Poor
53	53	100 pM	Poor
54	54	100 pM	Poor
55	55	1 nM	Poor
56	56	Toxic at 100 pM	Poor
57	57	100 pM	High
58	58	100 pM	Medium
59	59	100 pM	Medium
60	60	100 pM	Medium
61	61	100 pM	Poor
62	62	1 nM	Poor
63	63	1 μM	High
64	64	10 nM	High
65	65	100 pM	Medium
66	66	10 nM	Medium
67	67	100 pM	Medium
68	68	10 nM	Medium
69	69	1 nM	Medium
70	70	1 μM	High
71	71	100 pM	High
72	72	1 μM	High
73	73	100 nM	Medium
74	74	10 nM	High
75	75	100 nM	High
76	76	10 nM	High
77	77	1 μM	Medium
78	78	1 μM	Medium
79	79	100 nM	Medium
80	80	1 nM	Medium

Table S2. ClogP Values were generated (using Ligand Scout Software) to check the solubility and TPSA values of the compounds in the SAR study.

Compound	MolWt	Acceptors	Donors	Rings	Rot. Bonds	cLogP	Rel. TPSA
**3**	252.27	2	0	3	3	3.47	1.27
**4**	372.37	6	2	3	11	3.68	2.09
**5**	268.27	3	1	3	4	3.17	1.86
**6**	254.24	3	2	3	3	2.87	2.44
**9**	352.34	5	0	3	8	3.62	1.97
**10**	238.24	2	1	3	2	3.17	1.8
**11**	312.32	4	0	3	7	3.49	1.48
**12**	252.27	2	0	3	3	3.47	1.27
**14**	252.27	2	0	3	3	3.47	1.27
**17**	426.38	8	0	3	12	3.24	2.42
**18**	268.27	3	1	3	4	3.17	1.86
**19**	324.33	4	0	3	7	3.7	1.64
**20**	280.28	3	0	3	4	3.39	1.71
**23**	286.24	5	4	3	5	2.28	3.58
**24**	268.27	3	1	3	4	3.17	1.86
**26**	298.29	4	1	3	6	3.18	1.91
**27**	252.27	2	0	3	3	3.47	1.27
**29**	254.24	3	2	3	3	2.87	2.44
**30**	284.27	4	2	3	5	2.88	2.42
**32**	268.27	3	2	3	4	3.18	2.21
**33**	282.29	3	0	3	5	3.48	1.39
**36**	294.31	3	0	3	5	3.69	1.57
**37**	268.27	3	1	3	4	3.17	1.86
**38**	426.51	5	0	3	15	5.83	1.1
**39**	426.51	5	0	3	15	5.83	1.1
**40**	268.27	3	2	3	4	3.18	2.21
**41**	314.29	5	2	3	7	2.89	2.41
**50**	282.29	3	0	3	5	3.48	1.39
**61**	312.32	4	0	3	7	3.49	1.48
**63**	340.33	5	0	3	8	3.4	1.83
**67**	372.37	6	1	3	11	3.59	1.86
**70**	268.27	3	1	3	4	3.17	1.86
**76**	386.4	4	0	4	7	4.68	1.4

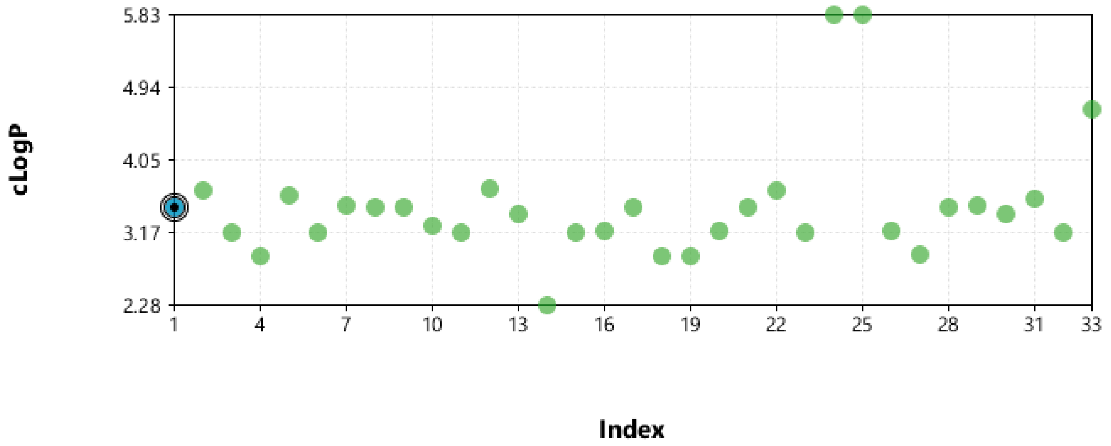

Figure S3. ClogP Values of compounds from table S2.

Table S3 The presence of more hydroxyl groups in the 14 flavonoid structure increases the neuro-differentiating activity.

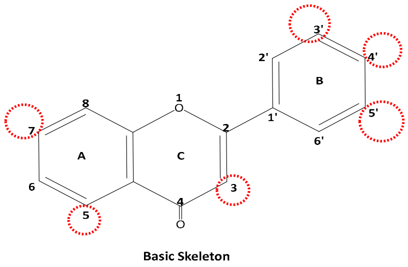

	Ring C	Ring A		Ring B		
**Compound**	C3	C5	C7	C3'	C4'	C5'
**4**	OCH_2CH_3	**OH**	**OH**		OCH_3	OCH_2CH_3
**5**			OCH_3	**OH**		
**6**		**OH**			**OH**	
**18**			**OH**		OCH_3	
**23**		**OH**	**OH**			**OH**
**24**	OCH_3				**OH**	
**29**					**OH**	
**32**	CH_3	**OH**	**OH**			
**37**						**OH**
**40**	CH_3		**OH**		**OH**	
**41**	OCH_3	**OH**	**OH**			OCH_3
**57**	CH_3	**OH**	**OH**			OCH_3
**64**		OCH_3			**OH**	
**70**		**OH**	OCH_3			

Table S4 Fewer hydroxyl groups in the 19 flavonoid structure give poor neuro-differentiating activity.

	**Ring C**	**Ring A**		**Ring B**		
**Compound**	C3	C5	C7	C3'	C4'	C5'
**3**					OCH_3	
**9**	CH_3		OAc		OAc	
**10**				**OH**		
**11**			OCH_3		OCH_3	OCH_3
**12**						
**14**			OCH_3			
**17**		OAc	OAc		OAc	OCH_3
**19**	CH_3		OAc			OCH_3
**20**		OAc				
**26**		OCH_3	OCH_3			
**27**						
**30**			OCH_3			
**33**					OCH_3	OCH_3
**36**	CH_3		OAc			
**38**		O-nPr	O-nPr		O-nPr	OCH_3
**39**		O-nPr	O-nPr		OCH_3	O-nPr
**50**						
**55**					$OCH_2(C_6H_6)$	
**61**			OCH_3			OCH_3

Table S5 The presence of one or more than one hydroxyl or methoxy substitution at the C5 and C7 position of Ring A and C4' position of Ring B of nine flavonoids shows higher activity; the absence of hydroxyl groups at the C5 and C7 position of 8 compounds showing lower activity.

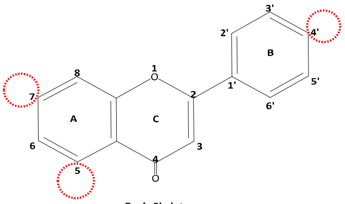

Basic Skeleton

	**Ring C**	**Ring A**		**Ring B**		
**Compound**	C3	**C5**	**C7**	C3'	**C4'**	C5'
**4**	OCH_2CH_3	**OH**	**OH**		OCH_3	OCH_2CH_3
**5**			OCH_3	**OH**		

**6**		**OH**			**OH**	
**18**			**OH**		OCH_3	
**23**		**OH**	**OH**			**OH**
**24**	OCH_3				**OH**	
**29**					**OH**	
**32**	CH_3	**OH**	**OH**			
**37**						**OH**
**40**	CH_3		**OH**		**OH**	
**41**	OCH_3	**OH**	**OH**			OCH_3
**57**	CH_3	**OH**	**OH**			OCH_3
**64**		OCH_3			**OH**	
**70**		**OH**	OCH_3			
**63**	OAc		OCH_3			OCH_3
**71**			OAc			
**72**		$OCH_2(C_2H_5)$	$OCH_2(C_2H_5)$		OCH_3	
**74**						
**76**	OAc				$OCH_2(C_6H_6)$	

Higher Activity	Lower Activity

Table.S6 Flavonoids **(4)**, **(5)** & **(23)** show antioxidant activity; position C5, C7, C3', and C4' hydroxylation exhibits solid antioxidant property.

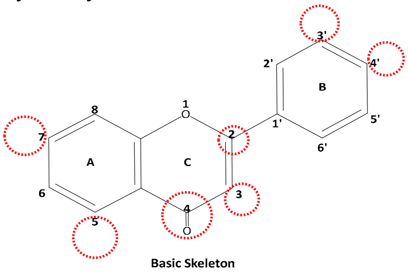

Basic Skeleton

	**Ring A**		**Ring B**	
**Compound**	C5	C7	C3'	C4'
**4**	**OH**	**OH**		OCH_3
**5**		OCH_3	**OH**	
**23**	**OH**	**OH**		

Table.S7 Compound **(5)** shows excellent anti-inflammatory activity compared to the other two compounds **(4)** & **(23)** because 3′-hydroxylation taking place along with 7-methoxylation greatly enhances anti-inflammatory properties

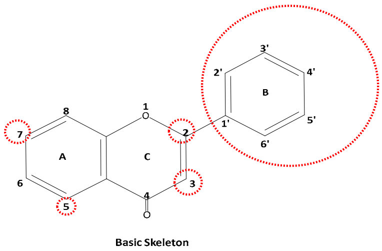

	**Ring A**		**Ring B**			
**Compound**	C5	C7	C2'	C3'	C4'	C5'
**4**	**OH**	**OH**			OCH_3	OCH_2CH_3
**5**		OCH_3		**OH**		
**23**	**OH**	**OH**	**OH**			**OH**

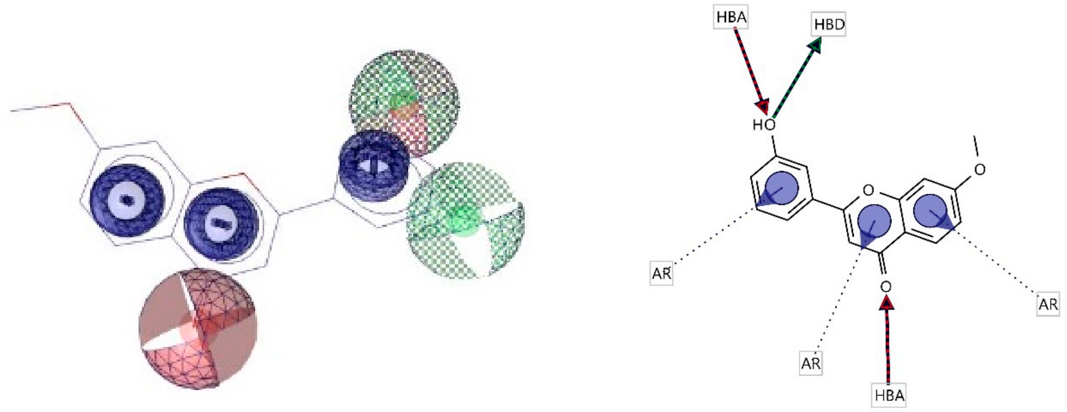

(5)

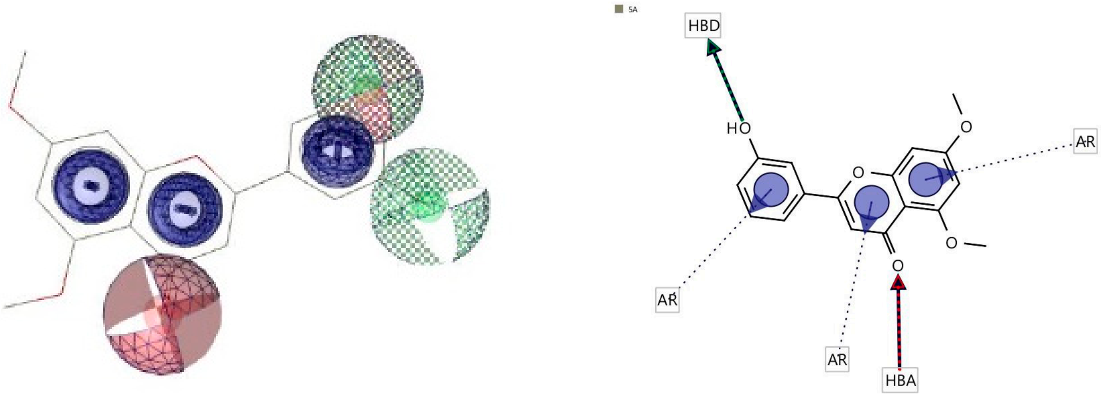

(5A)

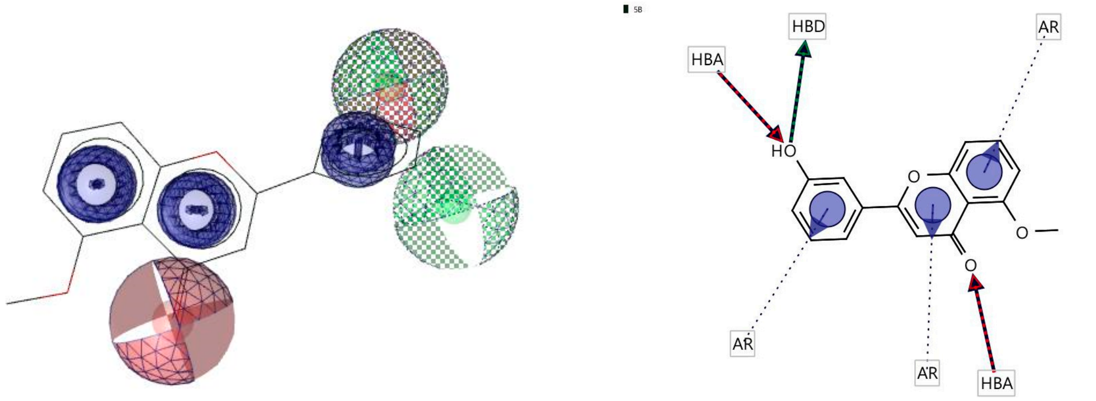
HBD
AR
HBA
HO
O
O
AR
AR
HBA

(5C)

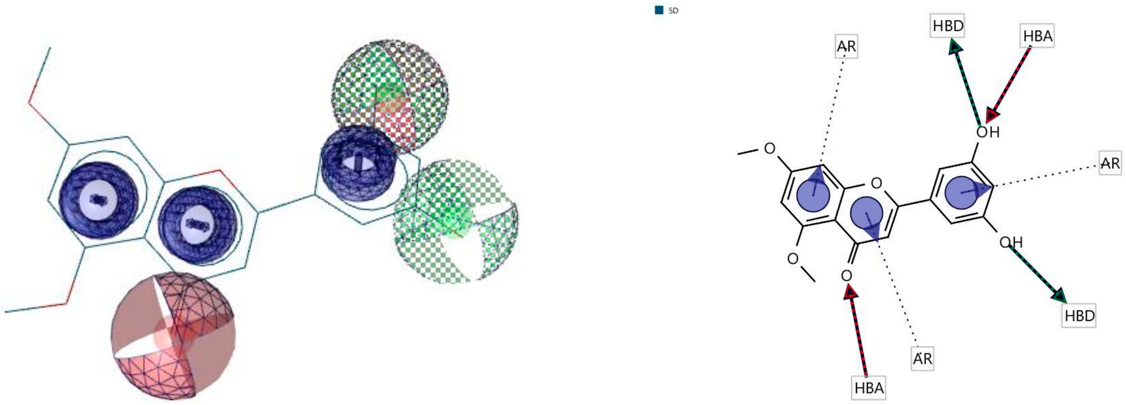
HBD
HBA
AR
OH
AR
O
O
OH
O
HBD
AR
HBA

(5D)

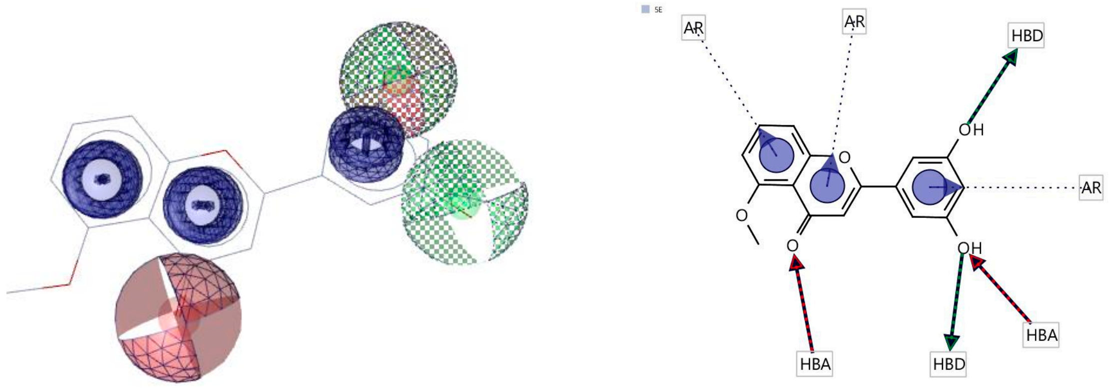
AR
AR
HBD
OH
O
AR
O
O
OH
HBA
HBD
HBA

(5E)

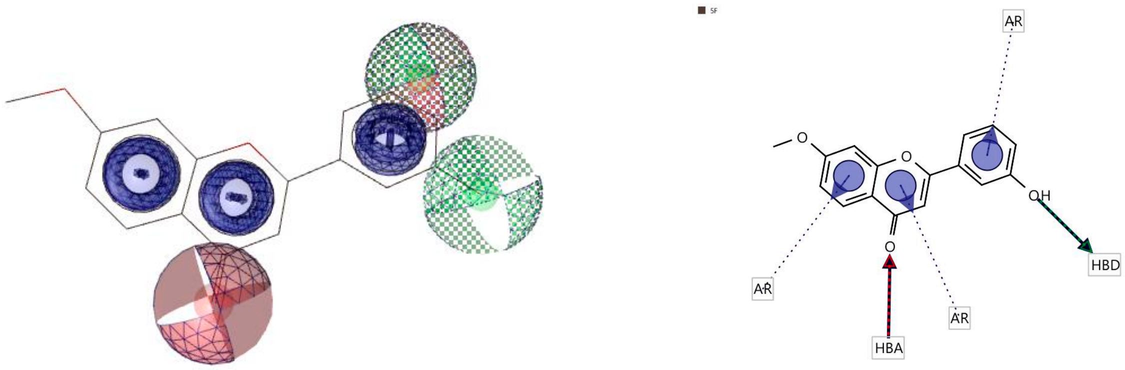
AR
O
O
OH
HBD
AR
O
AR
HBA

(5F)

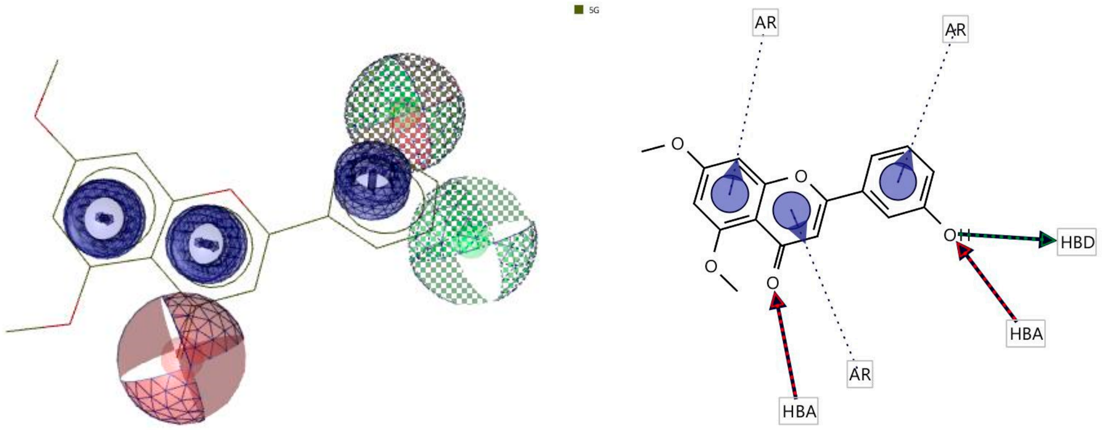
5G
AR
AR
HBD
HBA
AR
HBA

(5G)

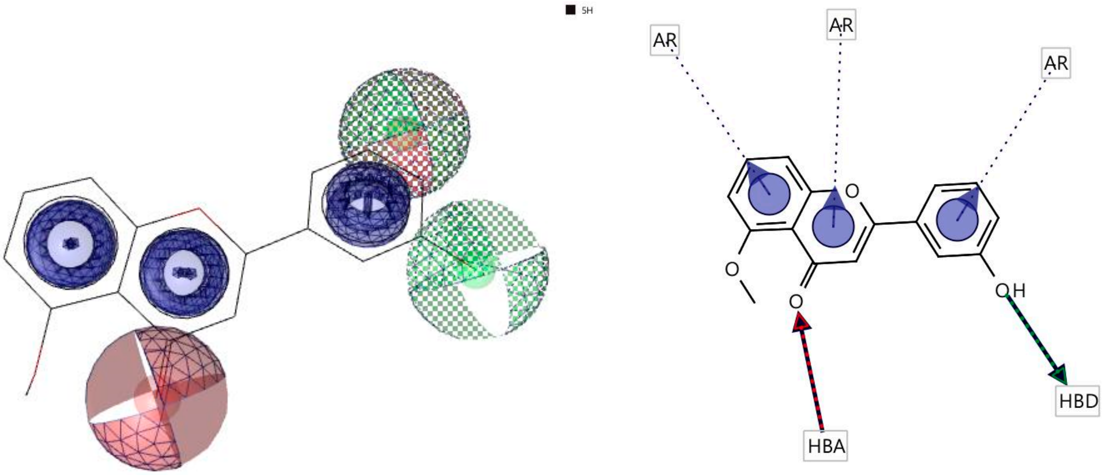
5H
AR
AR
AR
HBD
HBA

(5H)

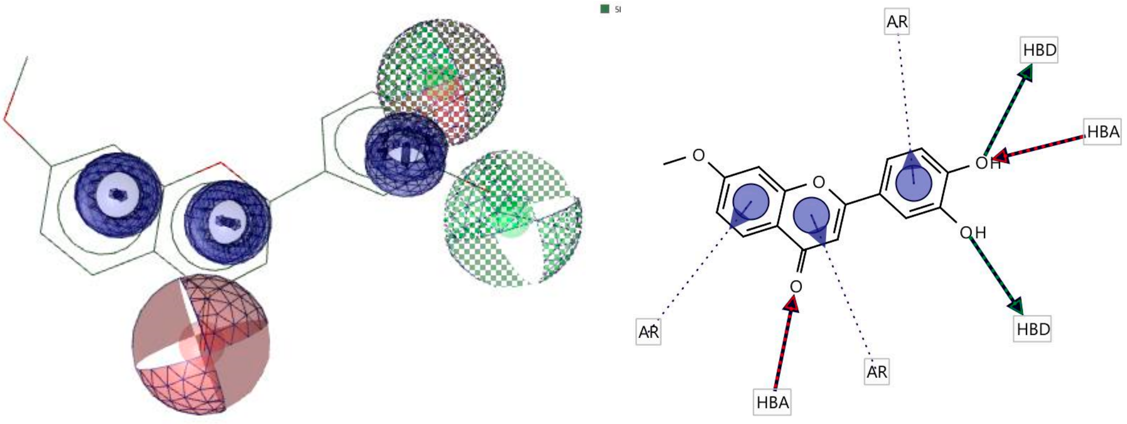
5I
AR
HBD
HBA
HBD
AR
AR
HBA

(5I)

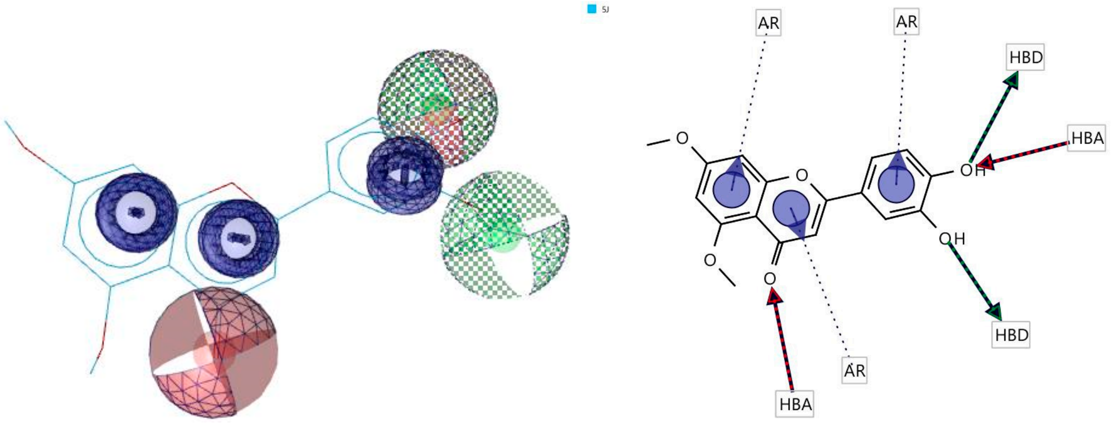
5J
AR
AR
HBD
HBA
HBD
AR
HBA

(5J)

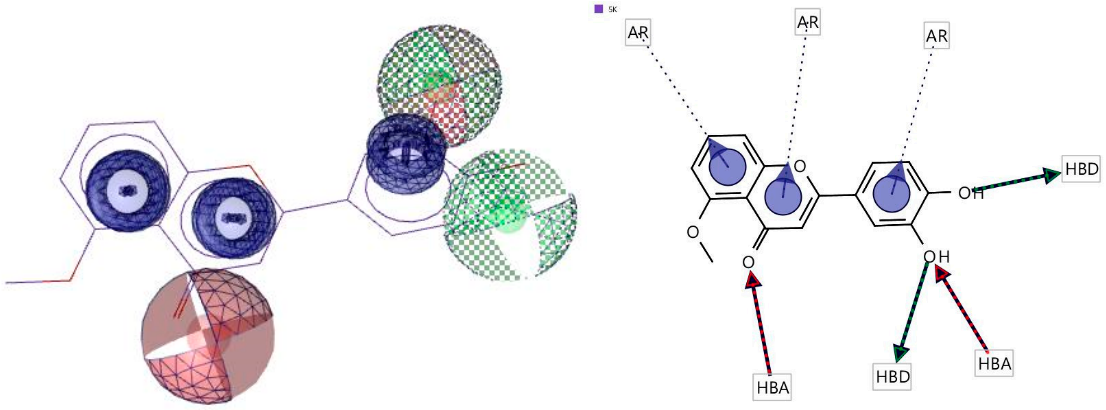
5K
AR
AR
AR
HBD
HBA
HBD
HBA

(5K)

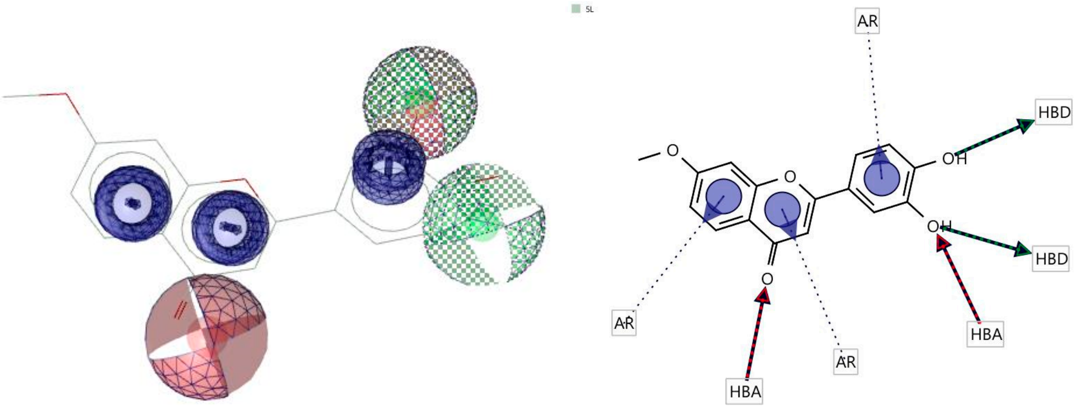
5L
AR
HBD
HBD
AR
HBA
AR
HBA

(5L)

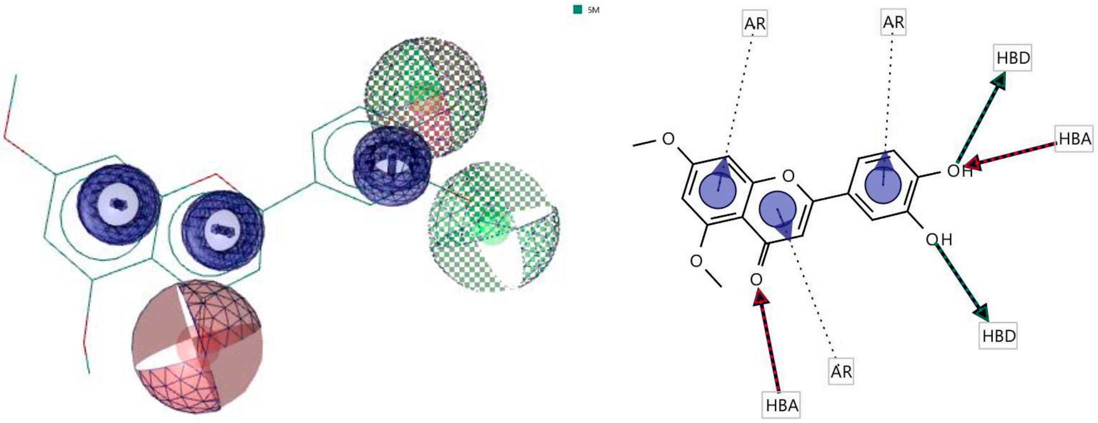
5M
AR
AR
HBD
HBA
HBD
AR
HBA

(5M)

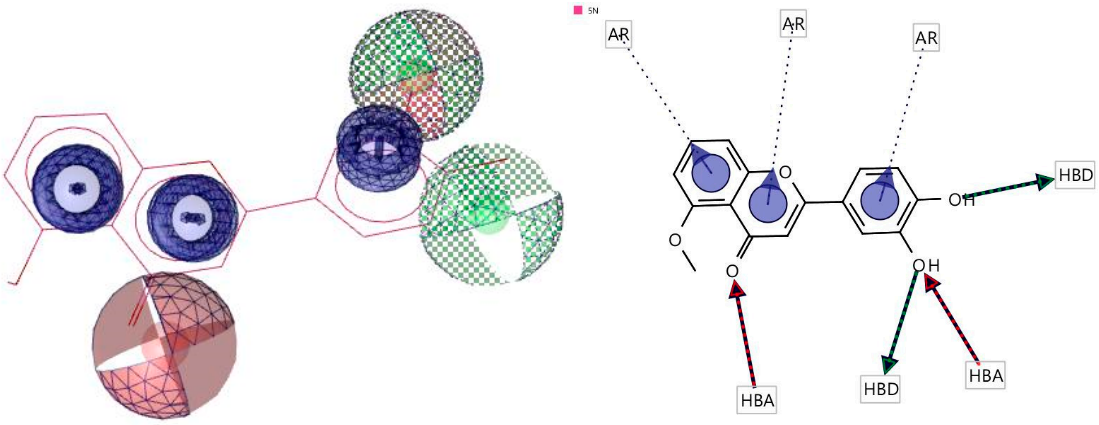
5N
AR
AR
AR
HBD
HBA
HBD
HBA

(5N)

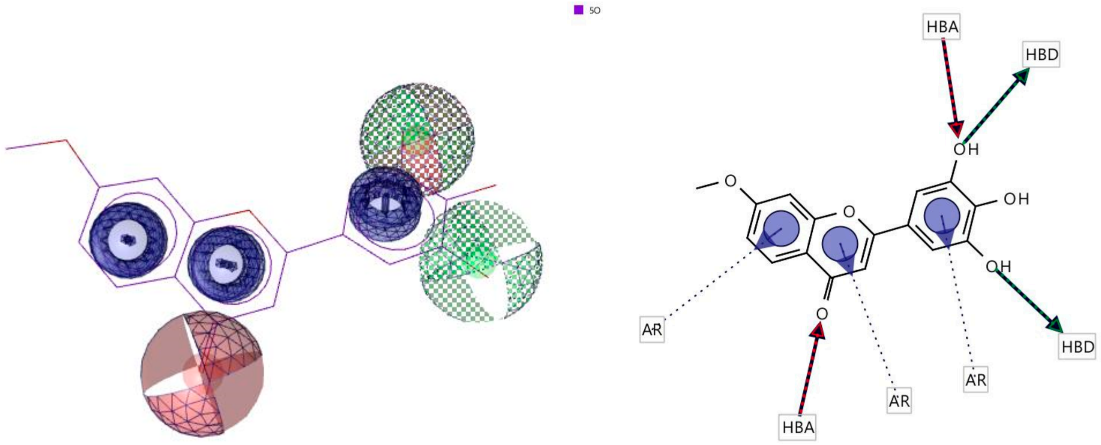

(5O)

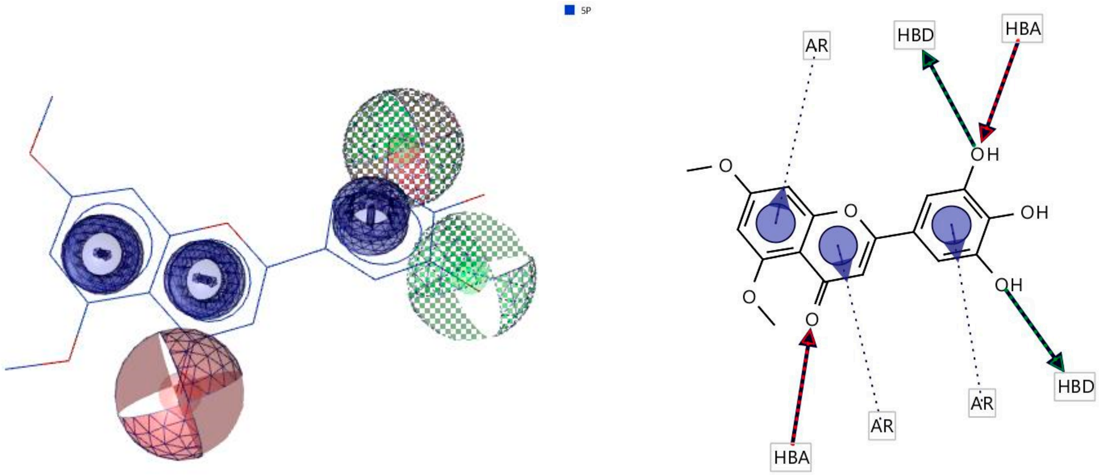

(5P)

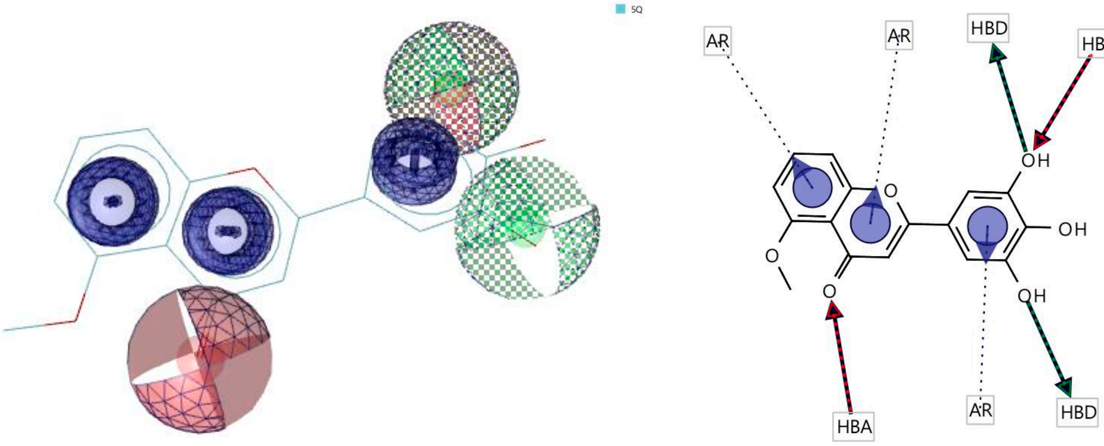

(5Q)

Figure S4. 3D and 2D representations of pharmacophoric features of the hypothetical compounds (5A – 5Q) used in 3D pharmacophore generation. Pharmacophoric features are color-coded with red, HBAs; green, HBDs; purple, AR.

(Method S4.2) Protocol for determining the highest non-cytotoxic dose of the compounds by the MTT assay ***(Provided by Axonova)***

1. SH-SY5Y cells were passaged and seeded at a density of 10,000 cells/well overnight.
2. The next day, the cells were treated with 200 μL of 100 pM to 10 μM of compounds for 48 h.
3. After the treatment time, the compound was removed, and 100 μL of 0.5 mg/mL of MTT dye was added for 2 h.

4. The MTT dye was removed, and 50 μL of DMSO was then added.
5. Absorbance was measured at 450 nm.
6. The percentage cell viability was calculated as per the equation below:

$$Cell\ viability\ (\%) = \frac{(Abs.\ of\ compound\ treated\ cells - Abs.of\ blank)}{(Abs.\ of\ control\ cells - Abs.of\ blank)}$$

Dose-response curve was generated for each compound, and the highest non-cytotoxic concentration was determined based on the selection criterion of 95 ± 5 % of cell viability.

(Method S4.3) Protocol for shortlisting the hits candidate with neurotrophic activity *(Provided by Axonova)*

1. SH-SY5Y cells were passaged and seeded at a confluency of 7,000 cells/well (500 μL) in a 24-well TPP plate.
2. Following 24h of plating, cells were treated with 10 μM of RA (retinoic acid) (500 μL) in 10% FBS (fetal bovine serum) media for 3 days.
3. RA-treated cells were then incubated with 500 μL of the highest non-cytotoxic concentration of the compounds, 10 μM of RA, 50 ng/mL of BDNF (Brain-derived neurotrophic factor) or vehicle in 1% FBS media for an additional 3 days.
4. Following the incubation periods, cells treated with RA and BDNF were refreshed with 10 μM of RA and 50 ng/mL BDNF for an additional 5 days. Cells treated with compounds were not refreshed with the compounds due to an observed cytotoxicity. Instead, these cells were incubated with 1% FBS media.
5. Images were captured on day 6 and day 10 of differentiation.
6. As a preliminary screening, the differentiation potential of the 80 compounds were compared to that of BDNF at day 6 of differentiation and were categorised as poor, medium, and high, as illustrated in Fig.S4.3.

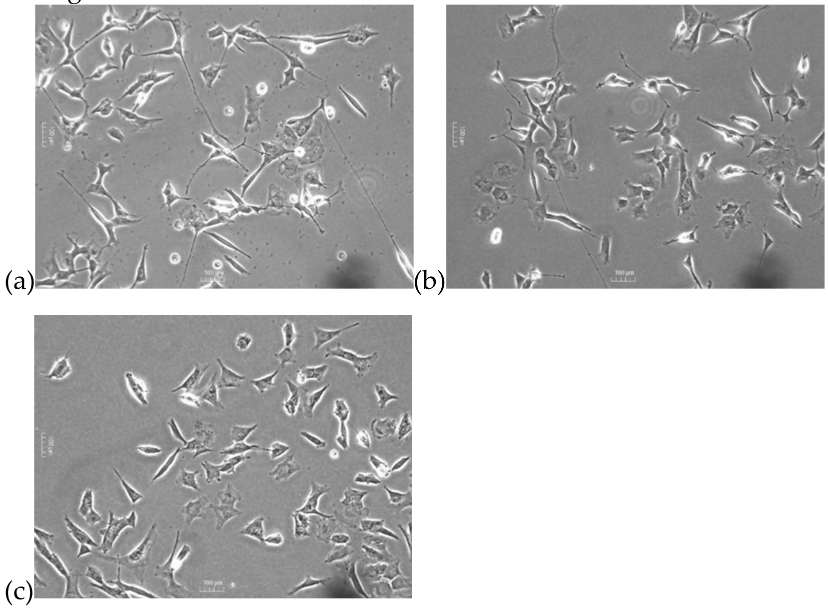

Figure S4.3. Representative figures of SH-SY5Y cells exposed to compound with **(a)** high, **(b)** medium and **(c)** poor differentiation abilities at day 6 of differentiation.

(Method S4.4) Protocol for assessing the neurotrophic and synaptogenic activities of the hit candidates by HCS *(Provided by Axonova)*

1. SH-SY5Y cells were passaged and seeded at a confluency of 3,000 cells/well (100 μL) in a 96-well TPP plate

2. Following 24h of plating, cells were treated with 10 μM of RA (100 μL) in 10% FBS media for 3 days.
3. RA-treated cells were then incubated with 100 μL of the highest non-cytotoxic concentration as well as 2 doses below the non-cytotoxic concentration of the compounds, 10 μM of RA, 50 ng/mL of BDNF or vehicle in 1% FBS media for an additional 3 days.
4. Following the incubation periods, cells treated with RA and BDNF were refreshed with 10 μM of RA and 50 ng/mL BDNF for an additional 5 days. Cells treated with compounds were not refreshed with the compounds due to an observed cytotoxicity. Instead, these cells were incubated with 1% FBS media.
5. Differentiation was stopped after 5 days.
6. The cells were washed with PBS, fixed with 50 μL of 3.7% formaldehyde for 15 mins and washed twice with PBS.
7. The cells were then permeabilised with 50 μL of permeabilisation reagent (0.1% Triton® X-100 in PBS) for 15 mins, followed by washing.
8. The cells were blocked with 50 μL of blocking solution comprising 1% BSA, 22.52 mg/mL glycine in PBST (PBS+ 0.1% Tween 20) for 30 min.
9. The cells were then incubated with the primary antibodies (diluted in 1% BSA in PBST) overnight at 4 °C, as per Table S4.4.
10. The next day, the cells were washed thrice with PBS and incubated with 1 μg/mL Hoechst and the secondary antibody (in 1% BSA in PBST), for 1 h at room temperature in the dark, as per Table S4.4.
11. After 1 h, the cells were washed thrice with PBS.
12. Images of the fluorescently tagged cells were captured and analysed by the HCS CX5.

Table S4.4 Primary and secondary antibodies are used for assessing neuronal differentiation and synaptogenesis

Marker	Primary antibody	Dilution	Secondary antibody	Dilution
Neuronal differentiation- neuronal marker	Rabbit anti-TUBB3	1:500	Donkey anti-rabbit Alexa Fluor 488	1:1000
Neuronal differentiation and synaptogenesis- dendrite marker	Chicken anti-MAP2	1:5000	Goat anti-chicken Alexa Fluor 594	1:1000
Synaptogenesis- presynaptic marker	Rabbit anti-Synaptophysin	1:200	Donkey anti-rabbit Alexa Fluor 488	1:1000
Synaptogenesis- post-synaptic marker	Mouse anti-PSD95	1:200	Goat anti-mouse Alexa Fluor 750	1:1000

(Method S4.5) Compound and insult treatment on differentiated SH-SY5Y cells *(Provided by Axonova)*

1. Following the differentiation of cells, the latter were pre-treated with the highest non-toxic dose of the hit compounds for 24 h.
2. The cells were then exposed to the Aβ 25-35, Aβ 1-40, Aβ 1-42, glutamate and H_2O_2 at the respective dose as listed in Table S4.5.
3. After 72 h, the parameters listed below were assessed.

Table S4.5 The concentration of insults used for assessing the neuroprotective activity of the hit compounds

Insult	Concentration	Treatment time

Aβ 25-35	40 μM	72 h
Aβ 1-40	35 μM	72 h
Aβ 1-42	20 μM	72 h
Glutamate	100 mM	72 h
H_2O_2	100 μm	72 h

(Method S4.5.1) Assessment of cell viability and ROS production *(Provided by Axonova)*

i. After 72 h, the cells were stained with 1 μg/mL of Hoechst 33342 (nuclear staining), 1 μg/mL of propidium iodide (staining of dead cells) and 5 μM of CellROX™ Deep Red Reagent (ROS marker).

ii. The plate was then read using the HCS CX5 at a magnification of 10x.

iii. The number of nuclei of live and dead cells and the fluorescence intensity of CellROX™ Deep Red Reagent were determined by the HCS.

(Method S4.5.2) Determination of neurite loss *(Provided by Axonova)*

i. Following extract and insult treatment, the cells were washed with PBS, fixed with 60 μL of 3.7% formaldehyde for 15 mins and washed twice with PBS.

ii. The cells were then permeabilised with 60 μL of permeabilisation reagent (0.1% Triton® X-100 in PBS) for 15 mins, followed by washing.

iii. The cells were blocked with 60 μL of blocking solution comprising 1% BSA, 22.52 mg/mL glycine in PBST (PBS+ 0.1% Tween 20) for 30 min.

iv. The cells were then incubated with the TUBB3 primary antibody (diluted in 1% BSA in PBST) overnight at 4 °C, as per Table S.4.5.2

v. The next day, the cells were washed thrice with PBS and incubated with 1 μg/mL Hoechst and the secondary antibody (in 1% BSA in PBST), for 1 h at room temperature in the dark, as per Table S.4.5.2

vi. After 1 h, the cells were washed thrice with PBS.

vii. Images of the fluorescently tagged cells were captured and analysed by the HCS CX5.

viii. The neurite length and number were then determined.

Table S.4.5.2 Primary and secondary antibodies used during the study

Marker	Primary antibody	Dilution	Secondary antibody	Dilution
Neuronal differentiation- neuronal marker	Rabbit anti-TUBB3	1:500	Donkey anti-rabbit Alexa Fluor 488	1:1000
Anti-inflammatory marker	Rabbit anti-Nrf2	1:1000	Donkey anti-rabbit Alexa Fluor 488	1:1000
Pro-inflammatory marker	Mouse anti-NFκB	1:1000	Goat anti-mouse Alexa Fluor 750	1:1000

(Method S4.5.3) Investigation of secretion of TNFα and IL-1β *(Provided by Axonova)*

i. Following extract and insult treatment, the supernatant was collected, and ELISA was carried out as per the manufacturer's protocol.

ii. The Greiner plate was coated with 100 μL of either TNFα (DY210-05) or IL-1β (DY201-05) capture antibody overnight.

iii. Following 3 times washing with wash buffer, the wells were blocked using 300 μL of reagent diluent for 1 h.

iv. The plates were washed thrice, after which 100 μL of sample or standard was added for 2 h.
v. After washing the plates 3 times, 100 μL of detection antibody was added for 2 h.
vi. Prior to and after the addition of 100 μL of Streptavidin-HRP for 20 mins, the wells were washed three times.
vii. A total of 100 μL of TMB solution was added to each well for 20 mins, after which 2N HCl was added to stop the reaction.
viii. The absorbance was measured at 450 nm, and the level of TNFα and IL-1β was calculated following the plotting of the standard curves (Fig.S.4.5.3)

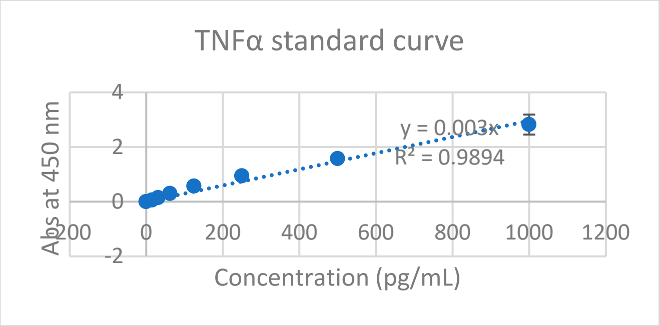

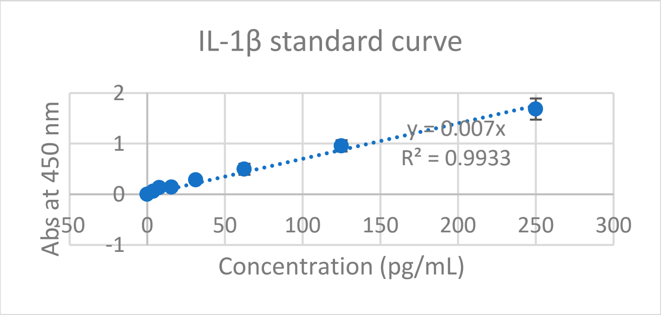

Figure S.4.5.3 (a)TNFα and **(b)** IL-1β standard curves.
